# Cas9-induced single cut enables highly efficient and template-free repair of a muscular dystrophy causing founder mutation

**DOI:** 10.1016/j.omtn.2023.02.005

**Published:** 2023-02-05

**Authors:** Stefanie Müthel, Andreas Marg, Busem Ignak, Janine Kieshauer, Helena Escobar, Christian Stadelmann, Simone Spuler

**Affiliations:** 1 Max-Delbrück-Center for Molecular Medicine in the Helmholtz Association (MDC), 13125 Berlin, Germany; 2 Muscle Research Unit at the Experimental and Clinical Research Center, a Cooperation Between the Max-Delbrück-Center for Molecular Medicine in the Helmholtz Association (MDC) and the Charité–Universitätsmedizin Berlin, 13125 Berlin, Germany; 3 Charité–Universitätsmedizin Berlin, Corporate Member of Freie Universität Berlin and Humboldt-Universität zu Berlin, Experimental and Clinical Research Center, 10117 Berlin, Germany; 4 Department of Biology, Chemistry and Pharmacy, Freie Universität Berlin, 14195 Berlin, Germany

**Keywords:** MT: RNA/DNA Editing, CRISPR-Cas9, gene editing, LGMD, stem cell therapy, mRNA

## Abstract

With thousands of patients worldwide, *CAPN3* c.550delA is the most frequent mutation causing severe, progressive, and untreatable limb girdle muscular dystrophy. We aimed to genetically correct this founder mutation in primary human muscle stem cells. We designed editing strategies providing CRISPR-Cas9 as plasmid and mRNA first in patient-derived induced pluripotent stem cells and applied this strategy then in primary human muscle stem cells from patients. Mutation-specific targeting yielded highly efficient and precise correction of *CAPN3* c.550delA to wild type for both cell types. Most likely a single cut generated by SpCas9 resulted in a 5′ staggered overhang of one base pair, which triggered an overhang-dependent base replication of an A:T at the mutation site. This recovered the open reading frame and the *CAPN3* DNA sequence was repaired template-free to wild type, which led to *CAPN3* mRNA and protein expression. Off-target analysis using amplicon sequencing of 43 *in silico* predicted sites demonstrates the safety of this approach. Our study extends previous usage of single cut DNA modification since our gene product has been repaired into the wild-type *CAPN3* sequence with the perspective of a real cure.

## Graphical abstract

**Figure:**
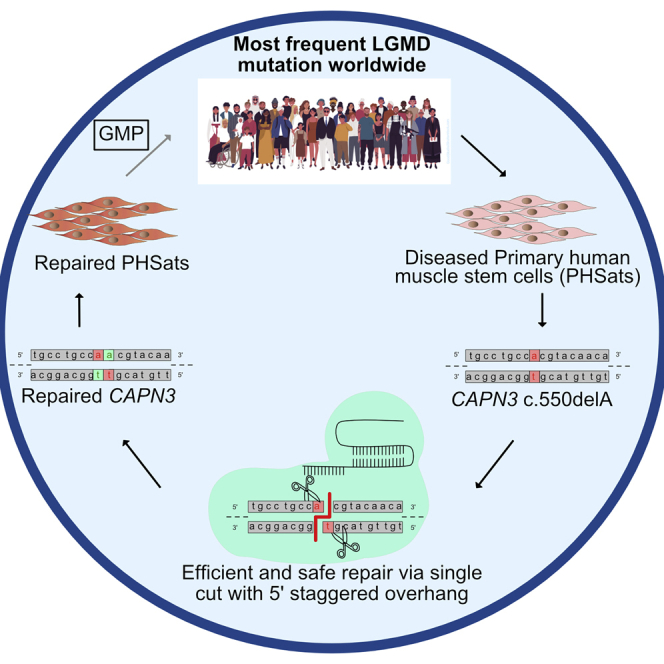

## Teaser

A precise, efficient, and safe gene repair with non-homologous end-joining after generation of a staggered SpCas9-mediated double-strand break is possible. We show this by repairing the most common founder mutation in LGMD2A *in vitro* giving the perspective for a real cure of this untreatable disease.

## Introduction

Limb girdle muscular dystrophies (LGMDs) are a group of nearly 30 monogenic diseases that result in muscle attrition and weakness, with subsequent loss of ambulation sometimes accompanied by respiratory and cardiac failure. There is no treatment, and no medication has yet been approved for intervention. The most frequent form is LGMD2A (or R1), which is caused by mutations in *CAPN3*, the gene encoding for the muscle-specific cysteine protease calpain 3. Close to 80,000 patients worldwide are affected by this autosomal-recessive gene defect. Disease onset is usually at the beginning of the second decade of life with severe muscle atrophy. Patients lose their ability to walk independently usually within 15 years.

Several hundred disease-causing mutations have been described for *CAPN3*. They are distributed across the entire length of the gene. One mutation, *CAPN3* c.550delA, is a founder mutation originating from the Eastern Mediterranean and is, with a reported prevalence of 1 in 144,000, the most common mutation of LGMD2A.^1^^,^^2^^,^^3^^,^^4^^,^^5^

Calpain 3 is a cysteine protease mainly expressed in muscle. Although already known for more than 30 years, the function of calpain 3 remains elusive. The protein undergoes an extremely rapid auto-degradation,^6^ which hampers biochemical analysis and the understanding of the pathomechanism of LGMD2A. Studies suggested that CAPN3 binds to titin/connectin, which is essential for sarcomere maintenance and remodeling.^7^^,^^8^ Identified targets of calpain 3 proteolytic function are fodrin,^9^ SERCA,^10^ and AHNAK.^11^ AHNAK is a giant protein, which is degraded upon expression of active calpain 3. Cells transfected with inactive calpain 3 C129S lose the ability to cleave AHNAK.^11^ Calpain 3 also has a non-proteolytic function in stabilizing critical Ca^2+^-handling proteins, whereby it contributes to the maintenance of Ca^2+^ homeostasis^12^^,^^13^^,^^14^ and gene regulation^15^^,^^16^ as shown in calpain 3 knockout mice.^17^ Several secondary assays were developed to monitor calpain 3 activity, but none have yet gained international gold standard. This is further impeded by the lack of functional antibodies for immunofluorescence.

As for other muscular dystrophies (MDs) no treatment for LGMD2A is available, although several studies are on-going to establish gene therapy approaches. Delivery of *CAPN3* cDNA via adeno-associated virus to supplement *CAPN3* gene expression was successful in mice and non-human primates.^18^^,^^19^^,^^20^ Furthermore, first gene editing studies in induced pluripotent stem cells (iPSCs) showed successful re-expression of calpain 3 after gene repair *in vitro* and transplantation of repaired cells *in vivo.*^21^

Cell-based therapies to treat MDs are an attractive approach with several cell sources that have been evaluated for their potency to restore skeletal muscle function.^22^^,^^23^ However, full regenerative myogenic potential has been demonstrated only for proprietary skeletal muscle stem cells, so-called satellite cells.^24^^,^^25^^,^^26^ Satellite cells are remarkable as they are quiescent but remain able to regenerate skeletal muscle even in old age. We developed techniques to isolate and expand satellite cell populations in culture, while preventing early end differentiation/senescence, and keeping the cell cultures free of fibroblasts.^27^^,^^28^ First data from preclinical tests in mice and rat demonstrate the safety and efficacy of these primary human satellite cell-derived muscle stem cells (PHSats).

Before autologous cell-based therapies can be used for LGMD patients, the disease-causing mutation must be repaired. The recent implementation of bacterial type II clustered regularly interspaced short palindromic repeats (CRISPR)-CRISPR-associated protein (Cas9) system has immensely increased the versatility of gene repair.^29^ Single-guide RNA (sgRNA) molecules identify specific sites within the DNA, where Cas9 produces DNA double-strand breaks (DSBs) 3 bp upstream of the protospacer adjacent motif (PAM).^30^ DSBs are repaired by the internal cell DNA repair machinery: Homology-directed repair (HDR) is a precise repair pathway that relies on sequence homologies of a donor DNA template. Therefore, it is the favored pathway to precisely repair disease-causing mutations. However, it is only active in dividing cells and suffers from inefficiency.^31^ Non-homologous or microhomology-mediated end-joining (NHEJ or MMEJ) are the most common repair pathways but usually considered to be imprecise, random, and unpredictable, rendering them unsuitable for precise gene correction. Yet, NHEJ functions in non-dividing cells and therefore, potentially, could be active in most cells in the adult human body.^31^

Conventionally, Cas9 generates blunt-end DSBs that are repaired by NHEJ randomly with Ku proteins.^32^ However, Cas9 can also generate staggered ends with 1–3 nt overhangs at the 5′ end, which triggers an overhang-dependent base-fill.^33^^,^^34^^,^^35^^,^^36^^,^^37^ It appears that the fraction of staggered ends is dependent on the sgRNA sequence.^36^ Staggered-end repair leads to a more predictable gene repair via NHEJ, but clinical relevance of this reframing mechanism has not yet been shown.

We were able to repair the mutation *CAPN3* c.550delA with Cas9-induced NHEJ leading to a high repair bias toward a one nucleotide insertion at the DSB in primary human muscle stem cells.

## Results

### LGMD2A/R1 cohort and characterization of CAPN3 c.550delA

**Figure 1:**
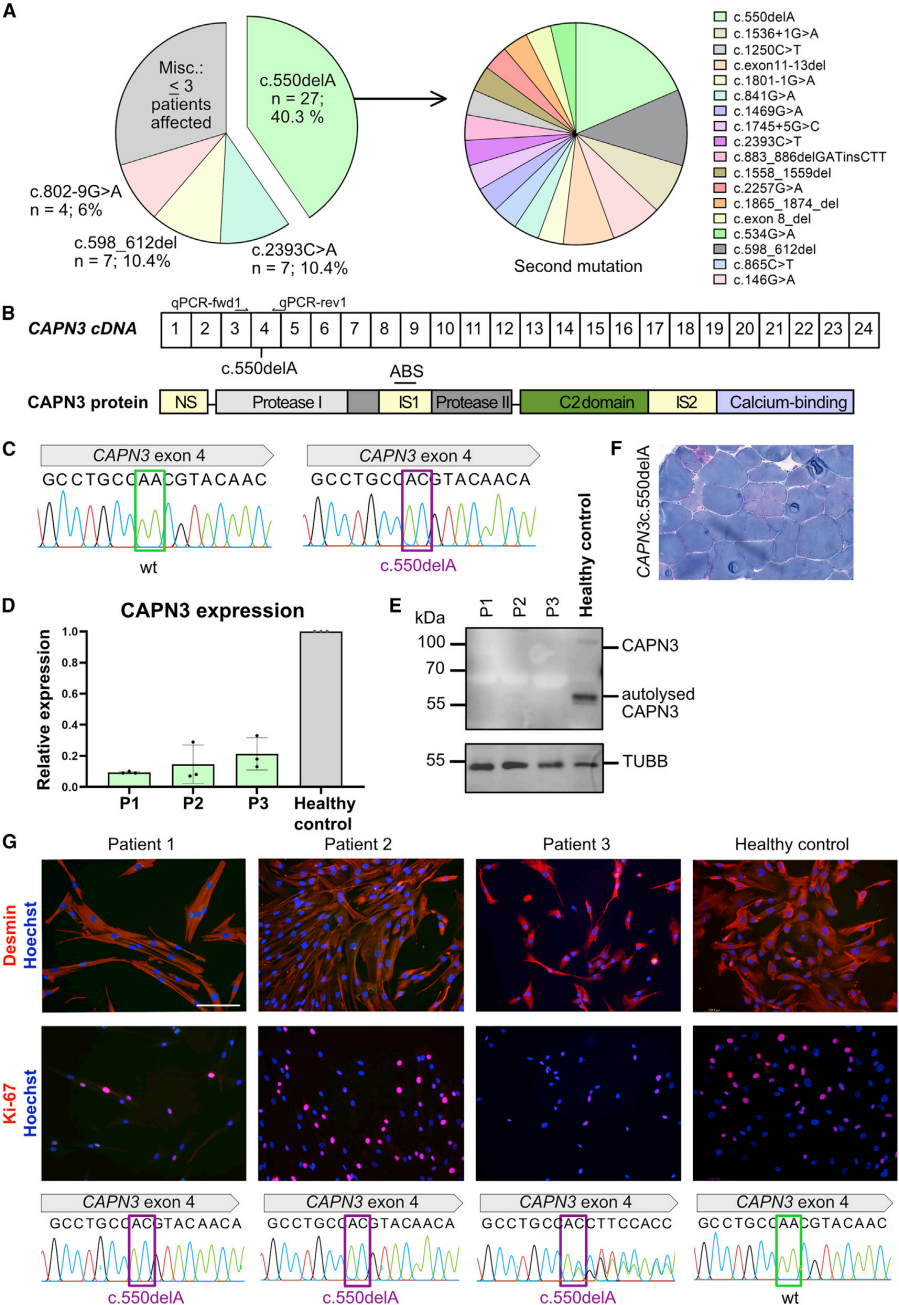
*CAPN3* c.550delA mutation (A) Left: frequency of *CAPN3* mutations in LGMD2A cohort (n = 67). *CAPN3* c.550delA is detected in 40% of patients. Right: second mutation with c.550delA. Five patients: homozygous for c.550delA, 22 are compound heterozygous. (B) Scheme of *CAPN3* cDNA (top; boxes represent exons) and protein structure (bottom) with site of c.550delA. NS, N terminus; IS1, IS2, insertion sequence; ABS, antibody binding site. (C) Sanger sequencing chromatogram of healthy (left) and *CAPN3* c.550delA (right). (D) qRT-PCR for *CAPN3* from three patients with LGMD2A and healthy control (Table S1). All patients carry *CAPN3* c.550delA which leads to degradation of mRNA. Patients 1 and 2 are c.550delA^+/+^, whereas patient 3 is c.550delA^+/−^. (E) CAPN3 protein detection in skeletal muscle tissue by western blot from the same patients (Table S1). Patients 1–3 do not show CAPN3. (F) Histology of skeletal muscle (*M. vast. lat.*) of a patient with *CAPN3* c.550delA. (G) Top: immunostaining of PHSats for the myogenic marker Desmin and the proliferation marker Ki-67 from patients 1–3 and healthy control. Nuclei were counterstained with Hoechst. Scale bar, 130 μm. At least 150 cells were counted. Bottom: DNA sequence analysis of the same patients and control.

We analyzed and quantified the *CAPN3* mutations in our cohort of LGMD2A/R1 patients (n = 67). *CAPN3* c.550delA was the most frequent *CAPN3* mutation^2^ (n = 27, 40%) (Figure 1A) followed by *CAPN3* c.2393C>A (n = 7) and *CAPN3* c.598_612del (n = 7). Five of the 27 *CAPN3* c.550delA patients were homozygous (*CAPN3* c.550delA^+/+^). In compound heterozygous patients, 15 different second mutations in *CAPN3* were identified (*CAPN3* c.550delA^+/−^; Figure 1A). The *CAPN3* c.550delA mutation is located in exon 4, which encodes for the protease I domain of calpain 3 (Figures 1B and 1C). The 1 nt deletion leads to a frameshift which induces a premature stop codon leading to loss of CAPN3 on mRNA and protein level (Figures 1D and 1E). Histologically, *CAPN3* c.550delA leads to typical features of a MD with pathological variation in fiber size, increase in connective tissue, necrosis, and regeneration (Figure 1F). We selected *CAPN3* c.550delA for gene correction because of its high frequency, its severity, and its particular relevance for treatment development of LGMD2A/R1.

To establish an autologous cell therapy approach, we generated PHSat populations from three patients with *CAPN3* c.550delA (two *CAPN3* c.550delA^+/+^, one *CAPN3* c.550delA^+/−^; Figure 1G; Table S1). Contaminating fibroblasts that may quickly overgrow myogenic cell populations, were eliminated as demonstrated by 100% Desmin-positive cells (Figure 1G). The proliferative capacity assessed by the cell-cycle marker Ki-67 was good with 20%–60% (Figure 1G). The *CAPN3* c.550delA mutant PHSats differentiated into mature myotubes (Figures S1A and S1B) and do not express CAPN3 protein (Figure S1C). These cells were used for further gene editing experiments.

### Reframing of CAPN3 c.550delA in iPSCs

To establish a gene editing approach, we first generated human iPSCs (hiPSCs) from a patient with homozygous *CAPN3* c.550delA. Reprogrammed cells expressed the pluripotency markers Oct4, Nanog, and Sox2, as wells as Tra-1-60, and differentiated into all three germ layers (Figures S2A–S2D).

**Figure 2:**
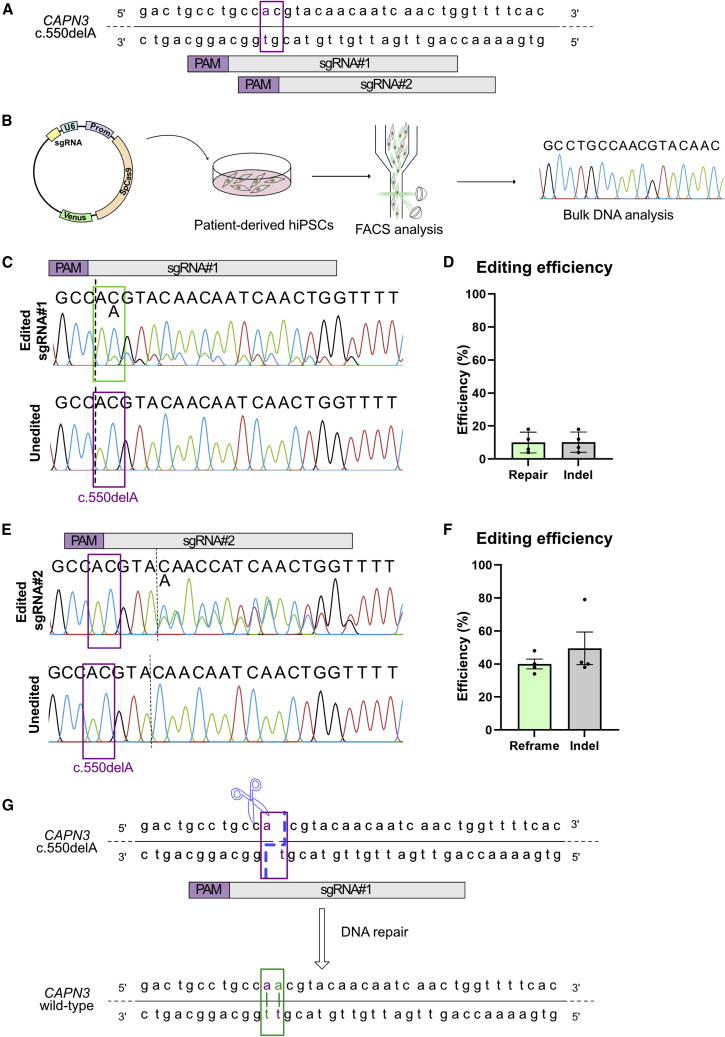
Efficient reframing by NHEJ (A) sgRNAs for c.550delA. The purple squares mark the position of the mutation. (B) Schematic overview of the experiment. Patient-derived hiPSCs were transfected with vector containing either sgRNA no. 1 or no. 2 and Cas9:T2A:Venus. Venus-positive cells were enriched with FACS and cells were processed for bulk DNA analysis. (C) Representative Sanger sequencing result for edited (top) compared with unedited (bottom) samples. Binding site of sgRNA is indicated on top. The purple squares mark the position of the mutation, dotted line represents the cutting site of SpCas9. sgRNA no. 1 leads to a frameshift by insertion of one adenine (green box). (D) Predicted editing efficiency of three biological repeats. The editing efficiency was predicted using ICE tool. In gray the indel frequency is quoted, green shows the repair by +1 insertion. (E) As in (C), but for sgRNA no. 2. (F) As in (D), but for sgRNA no. 2. Green bar shows the efficiency of reframing by +1 insertion. (G) Potential mechanism of precise reframing. Top: DNA sequence of *CAPN3* c.550delA; the purple square marks the position of the mutation, the dotted blue line indicates the SpCas9 staggered cutting model. Bottom: restored *CAPN3* wild-type DNA sequence after repair of the DSB.

We hypothesize that reframing with one nucleotide could recover the open reading frame (ORF), which might lead to re-expression of CAPN3 protein. We designed two mutation-specific sgRNAs (sgRNA no. 1 and no. 2; Figure 2A) and cloned them into a vector containing Cas9 from *Streptococcus pyogenes* with a T2A-Venus tag under the control of the CAG promotor. To assess reframing, we transfected patient hiPSCs carrying *CAPN3* c.550delA^+/+^, enriched Venus-positive cells via fluorescence-activated cell sorting (FACS) and analyzed the *CAPN3* c.550delA locus with Sanger sequencing (Figures 2B and S3). We found that both sgRNAs target the c.550delA locus. A heterogeneous cell population resulted from small insertions and deletions by NHEJ (indels) as demonstrated in the Sanger chromatogram by the occurrence of aberrant peaks 3′ prime of the cut site (Figures 2C and 2D). We could not detect any indels in unedited controls (Figures 2C and 2D). To identify indels, we used the ICE tool to facilitate decomposition of chromatograms.^38^ Both sgRNAs specifically generate a +1 insertion as a favored indel (Figures S4A and S4B), suggesting a successful reframing with both sgRNAs. In the case of sgRNA no. 1, the +1 insertion is occurring at the position of the mutation. This could restore the wild-type DNA sequence in *CAPN3* c.550delA if the inserted nucleotide is an A:T. To investigate this favorable outcome, we picked 48 single hiPSC clones from each biological repeat and analyzed the *CAPN3* c.550delA locus for both sgRNAs (Figures S4C and S4D). Indeed, we can detect the insertion of one A:T basepair following the cutting site. For sgRNA no. 1, 35% of singled iPSC clones have one (“het A/AA”) or both (“repair AA”) alleles modified with +1 A:T. For sgRNA no. 2, 50% of the clones for carry a +1 insertion.

We hypothesized, that reframing with a Cas9-induced staggered-end cleavage induces a duplication of the fourth nucleotide 5′ of the PAM. In the case of sgRNA no. 1 this leads to a precise repair of *CAPN3* (Figure 2G), whereas for sgRNA no. 2 the ORF of CAPN3 is reframed, but two new mutations are induced (T183R and Y184N; Figures S4E and S4F).

To confirm this cutting pattern for sgRNA no. 1, we amplified the *CAPN3* c.550delA locus and subjected the PCR product to digest with SpCas9 and sgRNA provided as ribonuclear particles. If a staggered-end cleavage is occurring, the nucleotide at the cut side is an adenine (A), whereas it is a cytosin (C) if the blunt-end model is applied (Figure S4G). We digested amplified *CAPN3* c.550delA and analyzed the restriction fragments (Figure S4H) with Sanger sequencing. As shown in Figure S4I, the last nucleotide at the cut side is an A, but not a C. This was verified with subcloning (Figure S4J) and confirms the above suggested staggered cutting pattern of SpCas9 at this position.

Since targeting with sgRNA no. 1 leads a precise repair of *CAPN3* c.550delA, we used this sgRNA for further experiments.

### Repair of CAPN3 c.550delA is highly increased by using SpCas9 mRNA

**Figure 3:**
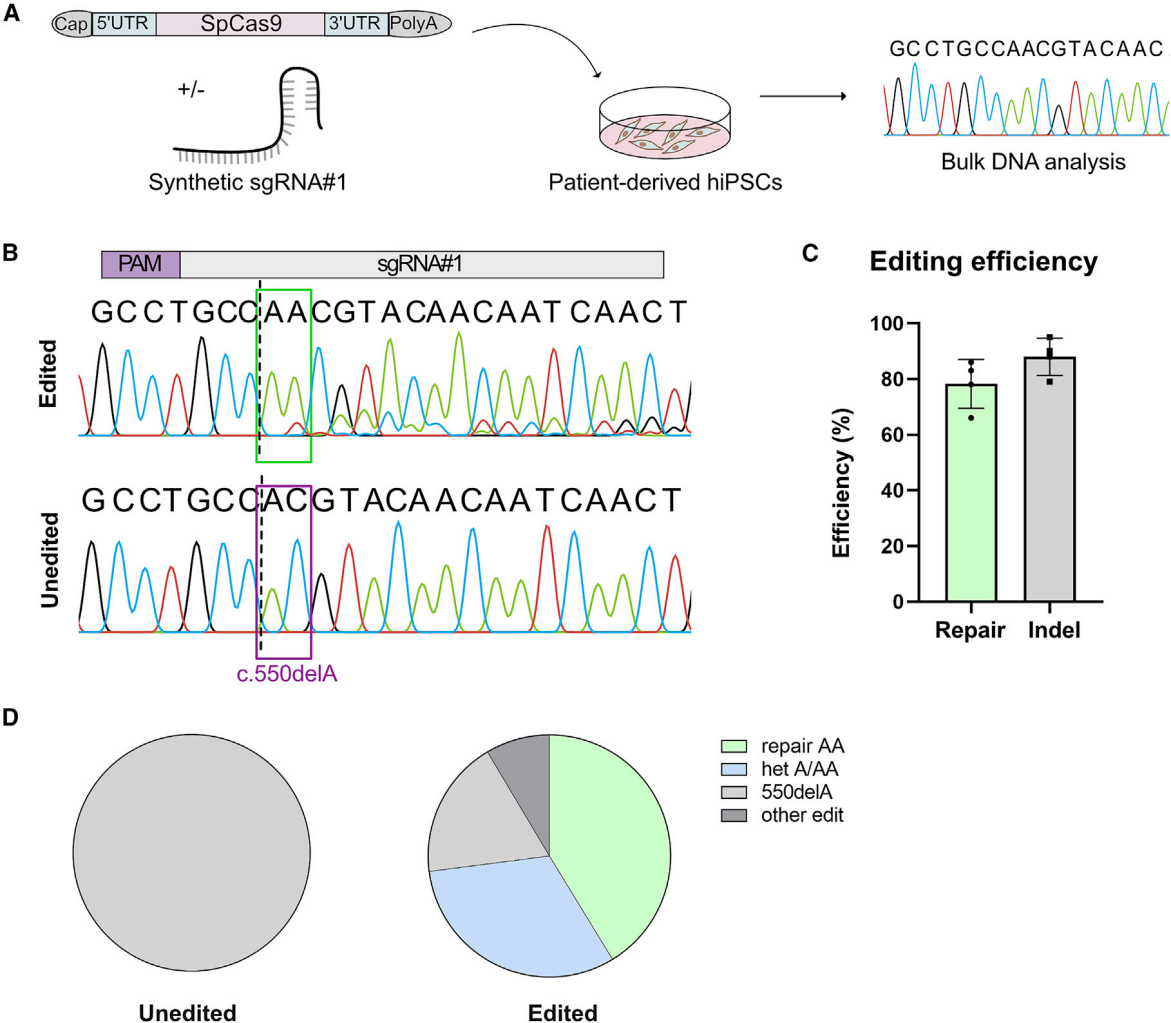
Efficient and precise reframing with mRNA in hiPCs (A) Schematic overview of the experiment. Patient-derived hiPSCs were transfected with sgRNA no. 1 and NLS-SpCas9-NLS mRNA. After expansion cells were processed for bulk DNA analysis. (B) Representative Sanger sequencing result for edited (top) compared with unedited (bottom) sample. Binding site of sgRNA is indicated on top, the purple square marks the position of the mutation, the dotted line represents the cutting site of SpCas9. sgRNA no. 1 leads to a frameshift by insertion of one adenine (green box). (C) Predicted editing efficiency of three biological repeats. The editing efficiency was predicted using ICE tool. In gray the indel frequency is quoted, green shows the repair by +1 insertion. (D) Verification of predicted editing efficiency by analysis of single hiPSC colonies. Single colonies were picked and the DNA sequence of *CAPN3* was analyzed with Sanger sequencing. For unedited samples 144 colonies and for edited samples 336 colonies in three biological repeats were analyzed.

Plasmid-based delivery of sgRNA no. 1 leads to a precise repair of *CAPN3* c.550delA, but regarding clinical translation, plasmid-based transfection is not optimal due to the risk of unwanted integrations into the host genome. Therefore, we decided to deliver Cas9 as mRNA (NLS-SpCas9-NLS) plus our designed, synthetic sgRNA no. 1 into patient hiPSCs (Figure 3A). Editing efficiency was analyzed using Sanger sequencing. We found that sgRNA no. 1 successfully targeted the c.550delA locus (Figures 3B and 3C), as demonstrated by the occurrence of indels 3′ prime of the cut site in the Sanger chromatogram. We could not detect any indels in unedited (mock and SpCas9 or sgRNA only) controls (Figures 3B, S5A, and S5B). To identify occurred indels and quantify the editing efficiency, we used the ICE tool. The overall predicted indel efficiency significantly increased from 7% with plasmid to 93% after mRNA delivery. Accordingly, the gene repair by insertion of a single adenine (+1 A) is also increased to 77% (Figures 3B and S5C). To verify the *CAPN3* repair by insertion of an A:T nucleotide*,* we picked 336 single hiPSC clones and analyzed the *CAPN3* c.550 locus. A homozygous repair was detected in 41% of the clones resulting in both alleles with wild-type *CAPN3* (repair AA). A heterozygous repair (“het AA/A”) with one allele edited and one allele unedited was found in 32% of the clones (Figure 3D). No sequence modification was seen in controls (Figures 3D and S5D).

In summary, 73% of the single-cell-derived hiPSC clones showed a successful repair of the mutation. We suggest that A:T is inserted via base-fill in at a 5′ overhang generated by SpCas9. This reframes and repairs the *CAPN3* locus.

### Efficient reframing of CAPN3 c.550delA in primary human muscle stem cells

**Figure 4:**
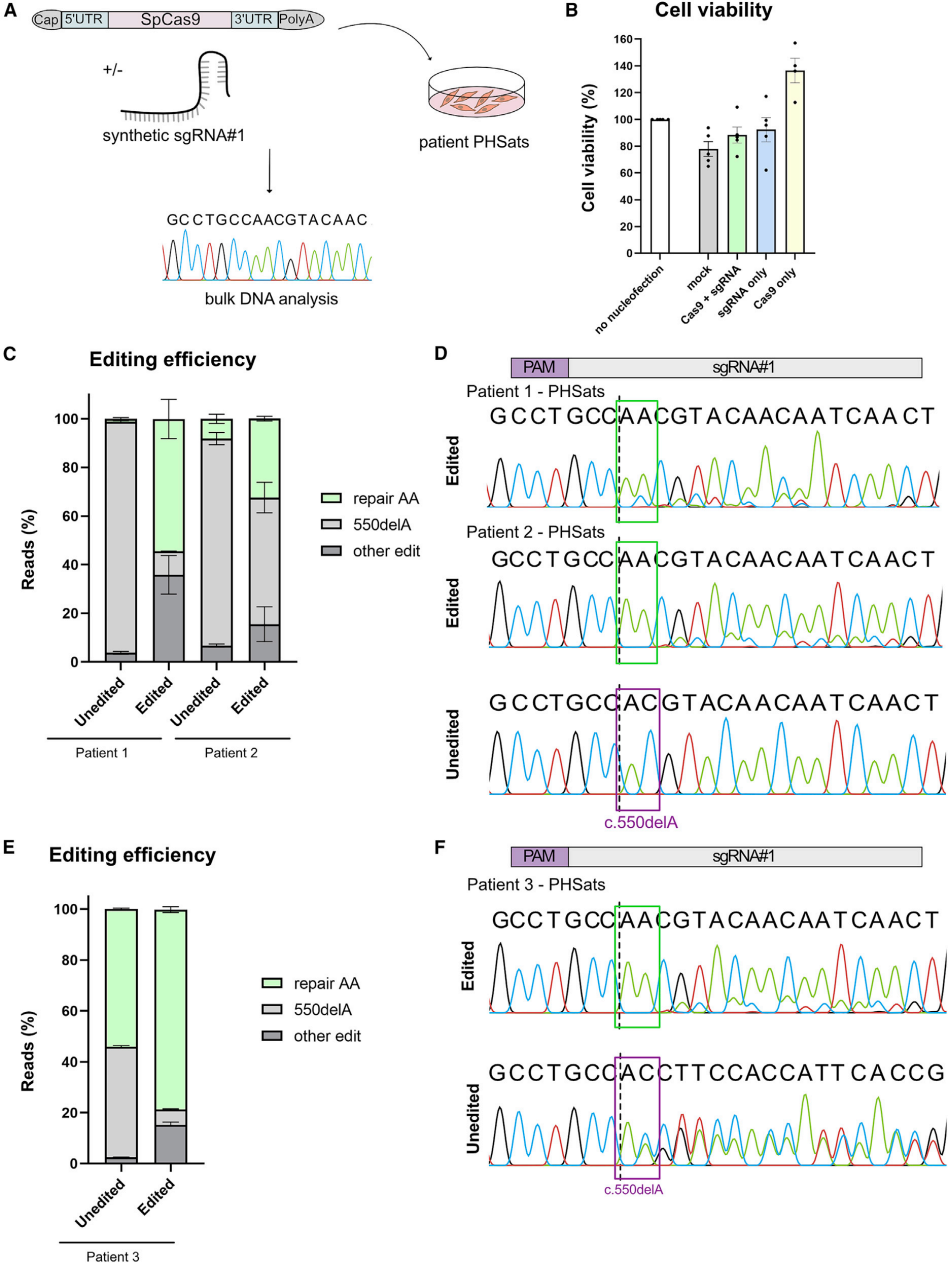
Efficient and precise reframing of *CAPN3* c.550delA in PHSats (A) Schematic overview of the experiment. Patient-derived PHSats were transfected with sgRNA no. 1 and NLS-SpCas9-NLS mRNA. After expansion cells were processed for bulk DNA analysis. (B) Survival of PHSats from patients 1 and 2 one day after nucleofection determined with MTT assay. (C) Editing efficiency as percent of reads determined by amplicon sequencing of three independent biological repeats for patients 1 and 2. (D) Sanger sequencing chromatograms for transfection of PHSats from patients 1 and 2 (homozygous *CAPN3* c.550delA^+/+^) with SpCas9 and sgRNA no. 1 (edited) compared with untransfected control (unedited). Binding site of the sgRNA is indicated on top; purple square, position of the mutation; dotted line, cutting site of SpCas9; green box, frameshift by insertion of one A:T. (E) Editing efficiency as percent of reads for patient 3 in two biological repeats. (F) Sanger sequencing result for transfection with SpCas9 and sgRNA no. 1 (edited) in PHSats from compound heterozygous patient 3 compared with untransfected control (unedited). Legend as in (D).

After having established an effective way to correct the *CAPN3* c.550delA mutation in hiPSCs, we aimed to repair the mutation in patient-derived PHSats. *CAPN3* c.550delA^+/+^ and *CAPN3* c.550delA^+/−^ PHSats were nucleofected with NLS-SpCas9-NLS and sgRNA no. 1 (Figure 4A). Viability after nucleofection was good, with similar rates of living, metabolically active cells compared with those that were not nucleofected (Figure 4B).

The correction of c.550delA to wild-type *CAPN3* DNA sequence was possible in all PHSat samples. In patients with homozygous mutations the repair efficiency due to a +1 indel bias (repair AA) was 55% for patient 1 and 33% for patient 2 (Figures 4C, S6B, and S6D). In the compound heterozygous samples from patient 3, we found that 79% of alleles carried the wild-type sequence after editing as opposed to 50% in unedited cells (Figure 4E). This is evident in the Sanger sequencing chromatograms (Figures 4D and 4F) and was confirmed by amplicon sequencing and Crispresso2 analysis (Figures 4C, 4E, and S7–S9). Amplicon sequencing revealed additional other edits, mainly small deletions around the cutting site that occur with low frequency (Figures 4C, 4E, S8, and S9).

These data confirm the successful gene repair of *CAPN3* c.550delA. In summary, the repair provides a highly efficient and precise DNA repair in PHSats from homozygous and heterozygous donors.

### No off-target editing at *in silico* predicted loci

**Figure 5:**
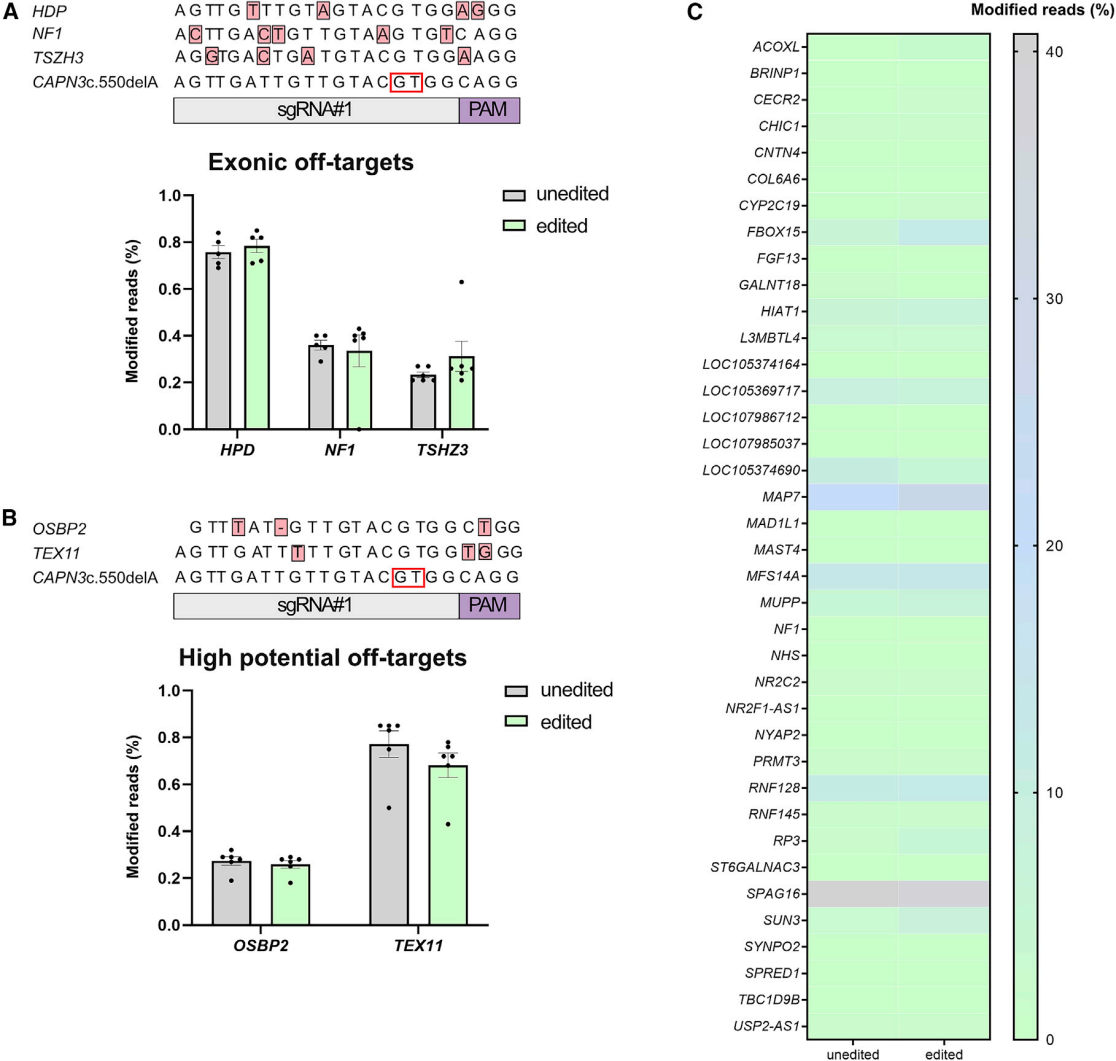
Off-target analysis (A) Modification of the predicted exonic off-targets. Percentage of modified reads within all reads at each locus after analysis with amplicon sequencing is plotted. No differences between edited and unedited control can be detected. Two biological repeats for patients 1–3 were analyzed. (B) Modification of the two off-targets predicted by CrispRGold with high potential of being targeted. No changes could be detected for any of the genes. Two biological repeats for each patient were analyzed. (C) Heatmap of amplicon sequencing result for modifications of 38 intronic off-targets predicted by CRISPOR and CrispRGold. Percentages plotted as in (A). None of the off-targets show a higher amount of modified reads in the edited vs. the unedited control. Two biological repeats from patient 2 were analyzed.

To assess the safety of gene editing using SpCas9, we performed an off-target analysis. First, we performed an *in silico* off-target prediction using CRISPOR.^39^ Off-target sites that differed from the on-target sgRNA sequence by up to four nucleotides were considered. Altogether, 112 off-targets were predicted (Table S2). We specifically were interested in the predicted exonic off-targets and analyzed the indel frequency with targeted deep sequencing. No modifications at the predicted sites were found in edited PHSats compared with unedited PHSats from patients 1–3 (Figure 5A; individual patients Figures S10A–S10C).

In addition, we used the *in silico* off-target prediction tool CrispRGold, which ranks the predicted off-target sites according to the potential (“risk score”) of being targeted.^40^ Twenty-four off-target sites with a “high” or “low” potential (or risk score) were predicted (Table S3). The highest potential of being targeted was assigned to off-target positions in the genes *OSBP2* and *TEX11*, which have sgRNA binding sites in the intronic region with three mismatches to sgRNA no. 1. Since they are more likely to be targeted, we analyzed the indel frequency in control and edited PHSats from patients 1–3 with targeted deep sequencing. Indels were detected, but no meaningful differences were found between unedited and edited cells at both sites (Figure 5B; individual patients Figures S10A–S10C). Finally, we analyzed 38 intronic off-targets, that were predicted to be targeted by both tools with amplicon sequencing from patient 1 (Figure 5C; Table S3). Again, we could not detect any off-target activity.

In summary, with our targeted NGS approach, we could not find any off-target editing in the predicted loci. This confirms that we can correct the most common LGMD2A mutation with high efficiency and precision without off-target modification at the analyzed sites.

### Myogenic and proliferative properties of PHSats after gene repair

**Figure 6:**
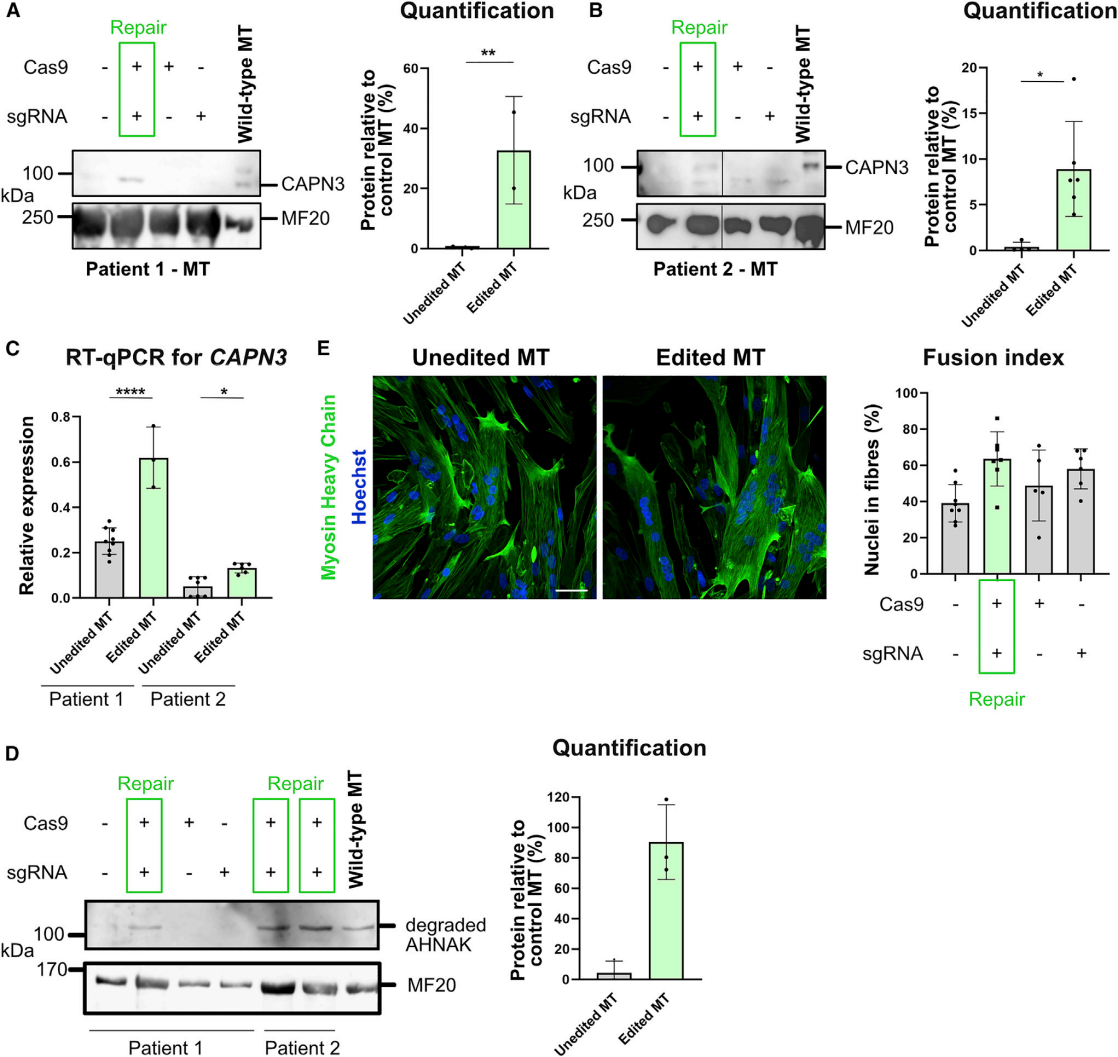
Functional recovery of CAPN3 expression (A) Left: western blot from myotubes (MTs) for CAPN3 after gene repair in patient 1. CAPN3 can be detected in edited MT. Right: quantification. Differentiated MTs that were edited show 30% protein recovery. The intensity of the CAPN3 band was normalized to MF20. Control MT are derived from healthy controls. Statistical analysis was done using unpaired t test; p < 0.0005; n = 2. (B) As in (A) for patient 2. Left: western blot. CAPN3 can be detected after editing. Lanes were run on the same gel, but noncontinuous. Right: quantification. Edited and differentiated MTs show 10% protein recovery compared with control MT. The intensity of the CAPN3 band was normalized to MF20. Control MT are derived from healthy controls. Statistical analysis was done using unpaired t test; p < 0.05, n = 5. (C) Detection of *CAPN3* expression by qRT-PCR. Consistent with the western blot results, *CAPN3* expression is recovered for both patients after editing. Relative expression was calculated to *GAPDH*. Statistics were done using one-way analysis of variance (ANOVA); p (patient 1) < 0.0001; p (patient 2) > 0.01), n = 3. (D) Western Blot from MTs for degradation of AHNAK after gene repair in patients 1 and 2. Degradation of AHNAK can be detected in edited MT. Control MT are derived from healthy controls. (E) Unedited or edited patient PHSats were differentiated into multinucleated MTs and stained for myosin heavy chain (MyHC). Nuclei were counterstained with Hoechst and fusion index for patient 2 was determined. Scale bar, 50 μm.

We showed that we can precisely, efficiently, and safely repair *CAPN3* c.550delA to the wild-type sequence. Next, we analyzed CAPN3 mRNA and protein expression after editing PHSats. CAPN3 expression is limited to terminally differentiated myotubes with almost mature sarcomeres. In myoblasts or in early myotubes, CAPN3 is not expressed yet. We generated mature myotubes^41^ from repaired PHSats from patients 1 and 2 (Figure S11A) and analyzed CAPN3 expression on protein and mRNA level. In both samples, CAPN3 protein expression could be demonstrated after gene repair (Figures 6A and 6B). CAPN3 was detected with up to 30% expression relative to control (Figures 6A, 6B, and S11C). *CAPN3* c.550delA leads to a frameshift and introduction of a premature stop codon. *CAPN3* mRNA is expected to be degraded by nonsense-mediated decay. This is consistent with low levels of *CAPN3* mRNA observed in our samples (Figures 1D and 6C). mRNA concentration is recovered after gene repair (Figure 6C).

### CAPN3 function normalized after gene repair

To test the functionality of repaired CAPN3, we analyzed the degradation of AHNAK, a target protein of calpain 3.^42^ Upon expression of CAPN3, AHNAK is degraded as shown in myotubes from healthy controls (Figure 6D). Only upon gene repair in both patients is the degradation band for AHNAK detectable, whereas no band is visible in unedited MT (Figures 6D and S11D). This confirms the functionality of CAPN3 in repaired PHSats.

We next investigated the myogenic and proliferative capacities of PHSats after gene correction. To determine if transfection and gene editing influence these factors, we analyzed the expression of the myogenic marker Desmin and the proliferation marker Ki-67 2 and 4 days after transfection (Figure S12A). Desmin remains high at both time points, proving that contaminating cells did not overgrow the edited population. After day 4, cells retain their proliferative capacity, as shown by Ki-67 expression. Commonly, no difference in the marker expression is detected between untransfected (unedited) and transfected cells. Furthermore, the fusion capacity was assessed based on the number of nuclei located in MyHC-positive fibers. After gene editing, fusion capacity and proliferation remained unchanged (Figures 6D and 6E). Together, the data confirm that transfection with SpCas9 mRNA or synthetic sgRNA is not toxic and has no adverse effects on PHSats.

### SpCas9 protein is quickly degraded after nucleofection

**Figure 7:**
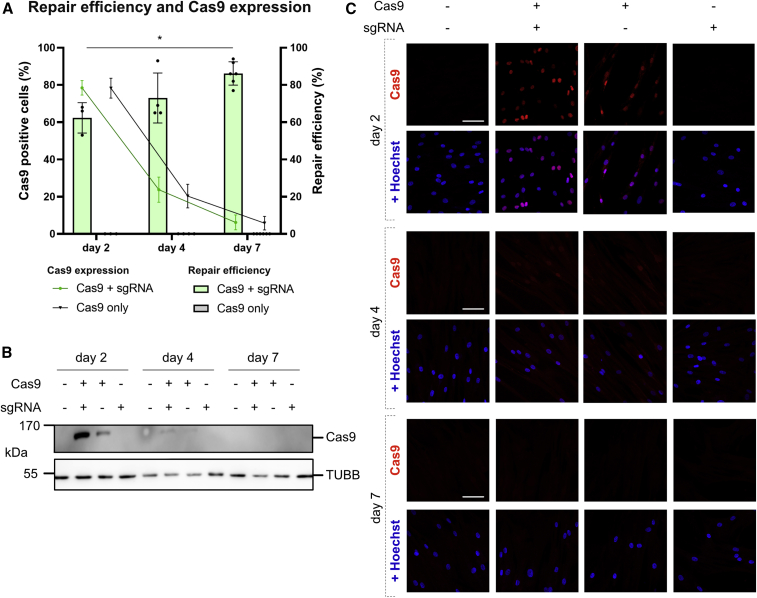
Rapid decrease in SpCas9 expression after transfection (A) DNA repair efficiency blotted with percent SpCas9-positive cells quantified with immunostaining. Repair efficiency was predicted using ICE tool. All experiments were carried out in two independent repeats from patient 1. Statistical analysis was done using two-way ANOVA; p < 0.01. (B) Western blot for SpCas9 expression at days 2, 4, and 7 after transfection. TUBB was used as loading control. (C) Immunofluorescence staining of SpCas9 (red) at days 2, 4, and 7 after transfection. Nuclei were counterstained with Hoechst. Scale bars, 50 μm. All experiments were done in PHSats from patient 1.

A prolonged presence of the nuclease Cas9 after successful editing could prevent translation into clinical application. We investigated the expression dynamics of mRNA-delivered Cas9 in PHSats 2, 4, and 7 days after transfection. Repair efficiency by +1 insertion was 62% 2 days after transfection and increased to 75% and 85%, respectively, at days 4 and 7 (Figures 7A and S13A). During the same time, SpCas9 expression decreased from 80% at day 2 to being undetectable at day 7 (Figures 7A–7C and S13B). This indicates that edited cells have a proliferative advantage over non-edited cells. The result also demonstrates rapid degradation of SpCas9.

### Repaired patient PHSats regenerate muscle

**Figure 8:**
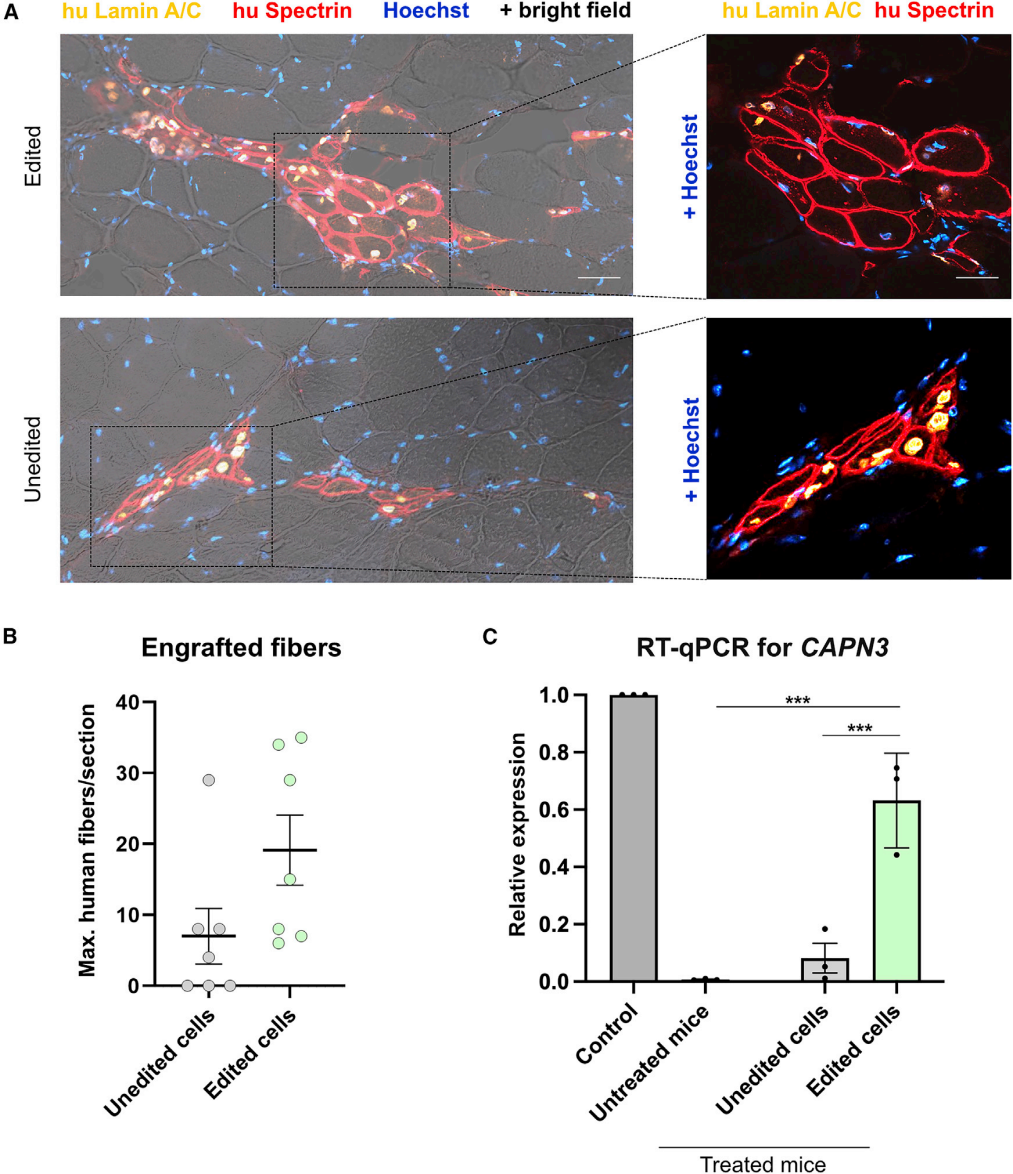
*In vivo* transplantation of PHSats into immune- and calpain 3-deficient mice (A) Representative image of immunofluorescence staining against human Lamin A/C and human Spectrin labeling donor nuclei and donor-derived human muscle fibers in injected TA muscles. Top: grafted muscle from edited cells. Bottom: graft formed by unedited cells. The right image depicts a close-up of the area of the transplanted muscle. Scale bars, 50 μM. (B) Quantification of engrafted myofibers. Spectrin-positive myofibers were counted. Grafts from edited cells are slightly bigger; p > 0.08, n = 7 animals; each quantification was done from a separate mouse. (C) Detection of *CAPN3* expression by RT-qPCR in grafted muscles. Control is C56BL/6; treated and untreated are C3KO-NSG. *CAPN3* expression is recovered after transplantation of gene edited PHSats. Relative expression was calculated to *GAPDH*. Statistics were done using one-way ANOVA; p < 0.01, n = 3.

To show the suitability of gene-corrected PHSats for cell therapeutic applications and test the *CAPN3* expression rescue *in vivo*, we transplanted repaired PHSats into irradiated anterior tibial (TA) muscles of immunocompromised and calpain 3-deficient mice (C3KO-NSG^21^; Figure S14A). PHSats from patient 1 were edited and injected, unedited cells were transplanted as control (Figure S14B). Edited and unedited cells integrate into the mouse muscle and give rise to human muscle fibers (Figure 8A). Grafts formed by edited cells contain 6–35 human muscle fibers and are slightly bigger than grafts from unedited cells (0–29 human muscle fibers) (Figure 8B; Table S4). Since no reliable antibody for CAPN3 immunostaining is available, we tested CAPN3 expression with qRT-PCR. Muscle tissue transplanted with edited cells shows a recovery of CAPN3 expression, whereas muscle tissue transplanted with unedited cells shows none (Figures 8C and S14C). Taken together, these data demonstrate that repaired PHSats from LGMD2A/R1 patients can generate myofibers *in vivo*.

## Discussion

We demonstrate that the most frequent mutation worldwide causing LGMD can be repaired in a template-free, efficient, and safe manner by classical SpCas9 delivered to primary human muscle stem cells by short-lived mRNA.

SpCas9 usually generates blunt-end DSBs with unpredictable editing outcomes. The specific indel bias to a +1 insertion of A:T that leads to the successful repair of *CAPN3* c.550delA could possibly be explained by a 5′ staggered overhang generated by SpCas9. For a staggered cut, the RuvC and HNH domains of Cas9 enable cleavage between nucleotides 3 and 4 on the target DNA and 4 and 5 on the non-target DNA upstream of the PAM.^43^ For inducing a clinically relevant staggered cut by Cas9, the sgRNA design is pivotal. The sgRNA must be positioned in a way that the mutation is located 4 bp upstream of the PAM and the following nucleotide that will be replicated is the same as the deleted base. With all these prerequisites fulfilled, the 5′ overhang triggers an overhang-dependent base-fill at position 4, which, then, produces a specific replication of the base at this position.^36^ We speculate that this mechanism is causing the reframing by +1 indel bias in this study and show that this mechanism can be used to repair clinically relevant deletion mutations.

The preference of the inserted nucleotide at the cleavage side and the cleavage mechanism are dependent on the local DNA sequence context. Studies show that, if one sgRNA is used for different cut sites, similar repair outcomes can be detected. This suggests that repair outcomes are non-random and depend on the target site sequence.^34^ It remains to be determined which nucleotides are inserted. We speculate that this might as well depend on the DNA sequence context and steric hindrance. However, the prediction of template-free SpCas9-mediated editing is possible^34^: SpCas9 was delivered as a plasmid in human cell lines leading to approximately 80% microhomology deletions and 10%–20% of 1 bp insertions. Using these data, Shen et al. created the machine learning tool inDelphi to predict editing outcomes at any given target site in different cell lines.^34^ Using our sgRNA as input, the tool predicts a +1 insertion bias with 60%–70%.

We confirm and extend the prediction in primary human muscle stem cells and iPSCs using mRNA-mediated SpCas9-delivery without reporter molecules and find a highly preferred 1 bp insertion while extensive off-target analysis revealed no editing events raising safety concern.

Reframing with insertion of one nucleotide was already shown before *in vivo* in mice and dogs for the *Dmd* gene.^44^^,^^45^ In those studies, the reframing restores the ORF leading to the production of functional, but partially truncated dystrophin, which is sufficient to rescue the disease phenotype.^44^^,^^45^ However, for rescue of *CAPN3* mutations a precise repair of the mutation is necessary since missense variants are not tolerated. We show here that reframing can be used to recover the wild-type DNA sequence that leads to expression of full-length protein and functional recovery *in vitro*. The systemic recovery of CAPN3 function with this editing approach *in vivo* still needs to be determined.

It is likely that other frequent and highly relevant frameshift mutations can be repaired using an indel bias resulting from predictable end-joining repair patterns. Since the mechanism relies on NHEJ, the repair is not cell-cycle dependent and more efficient than other CRISPR-Cas9-based gene repair options for this type of mutations. However, the compatibility for other frameshift mutations might be reduced due to the restrictions in sgRNA design since the mutation must be located 4 bp upstream of the PAM and the following nucleotide must be the same as the deleted one. Still, compared with other precise Cas9-based repair mechanisms like HDR or HITI, reframing by NHEJ is more efficient in post-mitotic muscle cells. Potential clinically relevant targets could be delA mutations in breast cancer-causing genes (e.g., *BRCA2* c.5578delA^46^ or *EP300* c.5099delA^47^; CinVar database www.ncbi.nlm.nih.gov/clinvar/).

Isolation and propagation of muscle stem cells from patients with MD is a challenge because the muscle tissue has partly been replaced by adipose and fibrous connective tissue. Contaminating fibroblasts tend to quickly overgrow the myogenic cells. We and others have developed methods that favor the cultivation of myogenic cells over co-isolated cell populations.^27^^,^^28^^,^^48^ Pure myogenic cultures are indispensable for autologous transplantations and our PHSats populations provide this purity.

In the case of autosomal recessive diseases, 50% of the protein dose suffices for being asymptomatic. Therefore, successful editing requires a correction rate of 50% or above. Exact quantification in our approach has been performed in isolated iPSC subclones, but in primary muscle stem cell populations clonal analysis is not possible. However, on-target amplicon sequencing yields reliable editing efficiency data. As we demonstrate here, up to 70% repair to control DNA sequence can be achieved for the *CAPN3* c.550delA founder mutation. After editing, expansion of the cell population is important to amplify healthy, non-mutated cells for autologous transplantation treatments. However, it is not readily evident whether the unedited, maybe less “stressed” cell population or the corrected PHSats have an advantage to expand in culture. Time course experiments show that, during 1 week after editing, the percentage of SpCas9-positive cells drops to almost zero, whereas the relative part of edited cells raises from 62% to more than 86%. This suggests that repaired cells divide and might even have an advantage to expand in culture.

However, a particular challenge could be the repair of compound heterozygous mutations. Most patients with *CAPN3* c.550delA carry the mutation in such a context. We achieved 79% wild-type AA in the sequencing reads, but 50% of the alleles obviously carry the second mutation. The question whether an efficient repair of one allele is sufficient for functional recovery remains unanswered. Simultaneous repair of two mutations in compound heterozygosity has not yet been shown but would be technically feasible.

With regard to a clinical application, we provided SpCas9 as mRNA apart from commonly used plasmids or ribonuclear particles. As we have shown before, nucleofection of mRNA provides a gentle and efficient delivery platform with optimal nucleofection efficiency resulting in homogeneous transgene expression in PHSats.^49^ Compared with plasmid-based delivery, mRNA strongly increased the editing efficiency and cell survival. Furthermore, mRNA delivery eliminates the risk of unwanted integrations of dsDNA into the host genome and can be directly translated in the cytoplasm without the need of entering the nucleus for transcription which is favored only during mitotic breakdown and reformation of the nuclear envelope.^50^ Ultimately, mRNA delivery provides faster and earlier expression kinetics compared with plasmid-based delivery.^51^ mRNA provides not only a fast bioavailability but is also degraded quickly. We show here that SpCas9 expression is almost undetectable 4 days after transfection. This reduces the risk of off-target effects. Due to the high specificity of sgRNA no. 1, such short expression of SpCas9 following mRNA-mediated delivery is sufficient to enable successful gene repair with high efficiency. Alternatively, SpCas9 could be provided as protein complexed with sgRNA in a ribonuclear particle. However, SpCas9 protein can easily denature, which could lead to difficulties to form loaded lipid nanoparticles (LNP).^52^ The feasibility of LNP-based delivery of mRNA has been shown impressively by the recent success of mRNA vaccines against SARS-CoV2^53^ and the first clinical trial of systemic CRISPR-Cas9 *in vivo* genome editing.^54^

So far, with our editing strategy we do not see off-target effects, as demonstrated by targeted analysis of 43 bioinformatically predicted off-target sites where no genome modifications could be detected. However, the off-target analysis needs to be expanded by additional cell-based off-target nomination methods such as GUIDE-Seq^55^ and by analysis of DNA translocations and large deletions.

We show that the efficient gene repair of *CAPN3* c.550delA is possible; however, the analysis of functionality of CAPN3 remains technically challenging in the *in vitro* setup. CAPN3 is not expressed in PHSats, but in terminally differentiated myotubes. *In vitro,* it is not possible to achieve a homogeneous differentiation of all PHSats. Therefore, not all PHSats terminally differentiate and express calpain 3. This explains the discrepancy between editing efficiency and expression of CAPN3 mRNA and protein.

Efficacy and safety of autologous intramuscular transplantation of repaired muscle stem cells in patients with MD needs to be evaluated in a first-in-human phase 1/2a clinical trial. We show here that transplantation of repaired PHSats from LGMD2A/R1 patients into the *M. tibialis anterior* of immunocompromised and calpain 3-deficient mice is successful and CAPN3 expression is rescued. In addition, it has been shown before that edited and healthy control cells successfully integrate into the muscle, build new muscle fibers, and repopulate the stem cell niche.^27^^,^^28^^,^^56^ Taken together, we confirm here that PHSats can be a valuable source for potential cell replacement therapies.

However, with only a limited number of edited cells available the approach may not gain sufficient functional recovery for patients, particularly in large muscles of the pelvic girdle. In addition, so far, no cell therapy approach was successful for MD. We would nevertheless consider repaired autologous PHSats as a valuable addition to the expanding portfolio of therapeutic strategies. Our cells are highly pure and the preclinical data show safety and efficacy of unedited cells. A first-in-human clinical trial using PHSats as an advanced therapeutic medicinal product is in preparation (Eudra-CT no. 2021-002004-13) and will ultimately proof the potential of PHSats in cell therapy.

In summary, we show an effective and precise repair to correct the most frequent LMD2A mutation *ex vivo*. Our approach is a promising source for autologous cell replacement therapies for LGMD2A/R1. The highly efficient correction of a *CAPN3* founder mutation by mRNA encoded SpCas9 sets the stage for further translation into clinics.

## Materials and methods

### Experimental design

A small muscle biopsy specimen (5 mm^3^) was taken under local anesthesia from *M. vast. lat.* or *M. deltoideus* of patients with confirmed mutation *CAPN3* c.550delA. After generation of PHSat cultures,^27^ cells were subjected to gene editing and subsequently expanded. Edited cells with appropriate controls were quantified and characterized regarding editing efficiency, CAPN3 expression, off-target editing, myogenic properties, and differentiation potential.

### Study approval

Research use of human material was approved by the regulatory agencies (EA2/051/10 and EA2/175/17, Charité Universitätsmedizin Berlin) and written informed consent was obtained from donors or legal guardians.

### sgRNA design

sgRNA was designed using CRISPOR^39^ with the input sequence “gtggttatagatgactgcctgccacgtacaacaatcaactggttttcaccaa” from *Homo sapiens* and a 20 bp -NGG PAM for SpCas9. The resulting sgRNAs were screened for having the SpCas9 cut site close to the position of the mutation (Table S5).

### Isolation and culture of primary PHSats

PHSats were isolated with hypothermic treatment as described previously.^27^^,^^28^ Cells were cultured in Skeletal Muscle Growth Medium (SMCGM) (Provitro, Berlin, Germany) enriched with supplement mix (Provitro) at 37°C in a humidified incubator with 5% CO_2_. For passaging, cells were washed with Dulbecco’s phosphate-buffered saline (DPBS) (Thermo Fisher Scientific, Waltham, MT) and detached with TrpLE Express (Thermo Fisher) at 37°C for 5 min.

### hiPSC generation, characterization, and cell culture

Patient hiPSCs were generated and characterized as previously described.^57^ In brief, we used PHSats from patient 2 (isolated as described above) and reprogrammed them using a Sendai Reprogramming Kit 2.0 (Thermo Fisher Scientific) on Matrigel-coated plates. Cells were kept in SMCGM + sodium butyrate (200 μM) + ascorbic acid (64 μg/μL) until the first hiPSC colonies with well-defined borders formed. The medium was changed to mTeSR1 (STEMCELL Technologies, Vancouver, Canada) and cells were transferred to 5% O_2_, 5% CO_2_, 37°C, 95% RH. For gene editing experiments, hiPSCs were cultured on Matrigel-coated plates (Corning, Corning, NY) in mTeSR Plus medium (STEMCELL Technologies) at 37°C in a humidified chamber with 5% CO_2_ and 5% O_2_. For passaging, cells were washed with DPBS and detached with 0.5 mM EDTA (Thermo Fisher Scientific). To achieve a single-cell suspension, hiPSCs were passaged with Accutase (Thermo Fisher Scientific).

### Cloning of SpCas9 and sgRNA expression plasmids

The plasmid HE_p4.1, which carries the expression cassette for sgRNA under the U6 promotor and SpCas9 under the CAG promotor was generated as described previously.^56^ For sgRNA cloning, the vector was digested with BplI and annealed sgRNA were ligated overnight at 16°C. Before ligation, sgRNA no. 1 and no. 2 (Table S5) were annealed using standard protocols. Ligated constructs were transformed into bacteria and positive clones were identified with Sanger sequencing.

### Lipofection of hiPSCs and FACS

hiPSCs were plated 1 day before lipofection with a density of 300,000 cells/9.6 cm^2^ in mTeSR Plus with 10 μM Rock inhibitor Y-27632 (Selleckchem, Houston, TX). Next day, cells were transfected using Lipofectamine 3000 Reagent (Thermo Fisher Scientific) according to the manufacturer’s instructions. Two days after transfection, cells were collected in PBS with 50% mTESR Plus, 0.1 mM EDTA, 10 μM Y-27632, and 100 μg/μL Primocin (Invivogen, Toulouse, France) and Venus-positive cells were sorted using a FACSAria cell sorter (BD Biosciences, Franklin Lakes, NJ) (Figure S3). Sorted cells were plated in mTeSR Plus with 10 μM Y-27632 and 100 μg/μL Primocin. The medium was changed next day to mTeSR Plus.

### Nucleofection of hiPSCs and PHSats

*hiPSCs*: hiPSCs were harvested as a single-cell suspension and, after washing with DPBS, resuspended in P3 Primary Cell Nucleofector Solution (Lonza, Basel, Switzerland) premixed with NLS-SpCas9-NLS (Aldevron, Fargo, ND) and/or sgRNA specific for *CAPN3* c.550delA (Synthego, Menlo Park, CA). For each nucleofection, 300,000 cells with 3 μg SpCas9 and sgRNA in a mass ratio of 1:0.67 was used in a 20 μL reaction (16-well nucleofection cuvette). For mock (unedited), cells were resuspended in P3 Primary Cell Nucleofector Solution. The cells were nucleofected using an Amaxa 4D Nucleofector (Lonza) with the CB-150 program. Afterward, 100 μL mTeSR Plus was added and cells were plated in mTeSR Plus with 10 μM Rock inhibitor Y-27632. The medium was changed next day to mTeSR Plus. *PHSats:* PHSats were harvested with TrypLE Express and, after washing with DPBS, resuspended in P5 Primary Cell Nucleofector Solution (Lonza) premixed with NLS-SpCas9-NLS (Aldevron) and/or sgRNA specific for *CAPN3* c.550delA (Synthego). For each nucleofection 150,000 cells with 3 μg SpCas9 and sgRNA in a mass ratio of 1:0.67 were used in a 20 μL reaction (16-well nucleofection cuvette). For mock (unedited), cells were resuspended in P5 Primary Cell Nucleofector Solution. The cells were nucleofected using an Amaxa 4D Nucleofector (Lonza) with the EY-100 program. Afterward 100 μL SMCGM was added, and cells were plated. The medium was changed next day.

### Isolation of single hiPSC clones

For singling hiPSCs, cells were seeded with 2,000 cells/9.6 cm^2^ in mTeSR Plus with 10 μM Y-27632 and kept in culture for 7 days. Single colonies were picked under a laminar flow hood in 96-well plates containing mTeSR Plus with 10 μM Y-27632. Next day, the medium was exchanged to mTeSR Plus. After 5–7 days, cells floating in the medium were harvested and used as PCR template to amplify and analyze the *CAPN3* c.550delA locus with Sanger sequencing.

### Viability after nucleofection

To analyze cell viability, CellTiter 96 Non-Radioactive Cell Proliferation Assay (Promega, Fitchburg, WI) was used according to the manufacturer’s instructions. In brief, after nucleofection cells were seeded with 3,000 cells/0.32 cm^2^ in 100 μL mTeSR Plus with 10 μM Y-27632. Next day, 15 μL of dye solution was added to each well and incubated for 4 h at 37°C. After adding 100 μL Stop solution, the absorbance was recorded at 570 nm with a 96-well plate reader.

### Genomic DNA extraction and sequencing analysis with ICE

Genomic DNA from PHSats was isolated with Agencourt AMPure XP beads (Beckman Coulter, CA) as described previously.^56^ For hiPSCs, genomic DNA was isolated with FlexiGene DNA kit (QIAGEN, Hilden, Germany) according to the manufacturer’s instructions. Target site amplification was done using Q5 High Fidelity DNA Polymerase (New England Biolabs, Ipswich, MT) and primers as indicated in Table S5. PCR products were purified using NucleoSpin Gel and a PCR Clean-up kit (Machery-Nagel, Düren, Germany). Sanger sequencing was performed with LGC Genomics (Berlin, Germany) and the resulting sequencing chromatograms were analyzed using an ICE CRISPR Analysis Tool (https://ice.synthego.com).^38^

### Cas9-mediated *in vitro* digestion of a PCR amplicon

We amplified *CAPN3* c.550delA from isolated genomic DNA with Q5 High Fidelity Polymerase (New England Biolabs) and purified the PCR product using NucleoSpin Gel and a PCR Clean-up kit (Machery-Nagel). The amplified PCR product was digested for 8 h at 37°C with 100 nM in-house produced SpCas9 (Addgene no. 69090) protein that was preincubated with sgRNA no. 1 (200 nM) for 10 min at 37°C. Digested fragments were separated with a 2% agarose gel and expected fragments with 173 and 239 bp were gel extracted with NucleoSpin Gel and a PCR Clean-up kit analyzed with Sanger sequencing. To verify the sequencing result, the 173 bp product was subcloned using a CloneJET PCR Cloning kit (Thermo Fisher Scientific) in *Escherichia coli* TOP10 (Thermo Fisher Scientific).

### Off-target prediction

We used CRISPOR^39^ and CrispRGold^40^ for off-target prediction with sgRNA no. 1 as sequence input. CrispRGold allows up to four mismatches and includes DNA bulges as well. Apart from identifying potential off-target sites, CrispRGold defines a type- and position-dependent mismatch-penalty matrix, which allows the calculation of the risk of each individual off-target.^40^ CrispRGold predicted 25 off-targets with high and low risk, as well as 880 sites that are unlikely to be targeted (lim→0). The latter were not analyzed further. CRISPOR predicted 112 off-targets with up to 4 mismatches. Most predicted off-targets are located in introns or intergenic regions and have 3 to 4 mismatches. A list of all high- and low-risk off-targets predicted with CrispRGold as well as all intronic and exonic off-targets predicted with CRISPOR can be found in Tables S2 and S3.

### Amplicon sequencing and CRISPResso2 analysis

Genomic DNA from PHSats was isolated as described above and target site amplification was carried out using Q5 High Fidelity DNA Polymerase (New England Biolabs) and primers as indicated in Table S6. PCR products were run on an agarose gel und gel purified using NucleoSpin Gel and a PCR Clean-up kit (Machery-Nagel). DNA concentration was measured using a Qbit fluorometer (Thermo Fisher Scientific) and the concentration was adjusted to 20 ng/μL. Next-generation sequencing (Amplicon EZ-service) was performed with GENEWIZ (Leipzig, Germany) using an Illumina MiSeq platform with 2 × 250 bp paired-end reads. Sequencing results were analyzed using CRISPResso2.^58^ The following parameters were applied: minimum homology for alignment to an amplicon: 60%; center of the quantification window (relative to the 3′ end of the provided sgRNA): −3; quantification window size (bp): 1; minimum average read quality (phred33 scale): >30; minimum single bp quality (phred33 scale): no filter; replace bases with N that have a quality lower than (phred333scale): no filter; exclude bp from the left side of the amplicon sequence for the quantification of the mutations: 15 bp; exclude bp from the right side of the amplicon sequence for the quantification of the mutations: 15 bp.

### Myotube differentiation and fusion index

*For CAPN3 detection:* The protocol was adapted from Guo et al.^41^ Cells were seeded on Matrigel-coated plates (Corning) in SMCGM with a density of 75 cells/mm^2^. When the cells reached confluency, myotube differentiation was induced by changing the medium to differentiation medium 1 (Dulbecco’s modified Eagle’s medium with 10 μg/mL insulin [Merck, Darmstadt, Germany], 500 μg/mL bovine serum albumin [BSA] [Merck], 10 ng/mL epidermal growth factor [Thermo Fisher Scientific], 50 μg/mL gentamycin [Merck]) for 7 days with ½ medium change every second day, followed by ½ medium change with differentiation medium 2 (Dif2) (Table S7) supplemented with 1 × G5 (Thermo Fisher Scientific) for 2 days. Afterward the medium was changed ½ with Dif2 (Table S7) without G5 for 2 days followed by 7 days in NbActiv1 (BrainBits, Springfield, IL) with ½ medium change every other day. Mature, multi-nucleated myotubes were used for downstream applications. *For fusion index:* cells were seeded in 8-well μ-slides with 12,000 cells/cm^2^. Upon reaching confluency, myotube fusion was induced by adding OptiMEM (Thermo Fisher Scientific) for 5–7 days. Cells were subjected to immunostaining as described below with antibodies in Table S8; ≥450 nuclei were counted per sample. To assess the fusion index the percentage of nuclei within myotubes (stained with MyHC) versus the total number of nuclei was calculated.

### Western blot

For myotubes, cells were harvested in CAPN3 lysis buffer (125 mM Tris [pH 6.8], 5% sodium dodecyl sulfate [SDS], 75 mM urea, 75 mM sucrose, 10 mM DTT, 10 mM EGTA, 10 mM EDTA) with cOmplete protein inhibitor (Sigma-Aldrich, St. Louis, MO). For tissue lysates, 5 × 50 μM tissue sections were cut and homogenized with a tissue homogenizer in CAPN3 lysis buffer. Protein concentration was determined using a BCA Protein Assay Kit (Thermo Fisher Scientific) and 20–30 μg protein from tissue or 25 μL cell lysates were diluted with 5× sample buffer (350 mM Tris-HCl, 30% glycerol, 10% SDS, 600 mM DTT, and 0.05% bromophenol blue) and boiled for 10 min at 96°C. Samples were run on 8%–16% gradient Tris-glycine acrylamide gel (Thermo Fisher Scientific) and blotted with SemiDry or Wet Transfer System (BioRad, Hercules, CA) on a nitrocellulose membrane (GE Healthcare, Chicago, IL). The membrane was blocked in 3% BSA for 1 h, incubated with primary antibody (Table S8) overnight at 4°C and HRP-conjugated secondary antibodies at RT for 1 h. The membrane was developed with SuperSignal West Dura Extended Duration Substrate (Thermo Fisher Scientific) and imaged using a VWR CHEMI only system (VWR, Radnor, PA). Quantification was done using Fiji.

### RT-PCR and qPCR

Total RNA was isolated with TRIzol (Thermo Fisher Scientific) following the manufacturer’s instructions. cDNA was synthesized from 500 ng RNA using a QuantiTect Reverse Transcription kit (QIAGEN). qPCR was performed using KAPA SYBR FAST qPCR Master Mix Universal (Sigma Aldrich) and measured using BioRad CFX (BioRad). Data were evaluated with the 2-ΔΔCT method^59^ with GAPDH as reference gene. Primers for qPCR are indicated in Table S6.

### Animal experiments

Animal experiments were performed under the license no. G0223/20 (LaGeSo Berlin, Germany). Capn3Gt(OST141731)Lex; NOD.Cg-Prkdcscid Il2rgtm1Wjl/SzJ were kindly shared by Rita Perlingeiro, Unniversity of Minnesota, USA. Breeding was done under the license no. G0337/19 (LaGeSo Berlin). Animals were kept at our specific pathogen-free animals facility with free access to food and water, provided with a hiding place and nest material. Hygienic monitoring was done according to FELASA recommendations.

### Intramuscular cell transplantation

Edited PHSats used for transplantation were 99% Desmin, 48% Ki-67+, and showed 81% +1 A at the position of the mutation (Figure S14B). Unedited control PHSats were 99% Desmin+, 30% Ki-67+ and showed no editing (Figure S14B). Focal irradiation of the recipient hindlimb was performed 2 days before cell transplantation as described before using a CyberKnife image-guided robotic radiosurgery system.^27^^,^^28^^,^^56^ For transplantation of PHSats, mice were placed under isoflurane anesthesia. After shaving the skin area over the TA muscle, 11 μL cell suspension with 1 × 10^5^ cells in sterile PBS +2% FBS was injected into the medial portion of the TA muscle as described before.^28^^,^^56^ Mice were sacrificed 21 days after cell transplantation. TA muscle was cryopreserved in liquid nitrogen-chilled isopentane mounted in gum traganth and stored at −80°C.

### Immunostaining

*Cells:* Cells were plated in 8-well μ-slides (ibidi, Gräfelfing, Germany) (3,000–12,000 cells/cm^2^). One day after or at a given time point, cells were fixed with 3.7% formaldehyde (Sigma-Aldrich) for 10 min at room temperature (RT). Cells were permeabilized using 0.2% Triton X-100 (Sigma-Aldrich; except Tra-1-60 staining) for 5 min and blocked with 5% BSA (Carl Roth, Karlsruhe, Germany) for 1 h at RT. *Tissue:* 6 μM cryosections were cut with a Leica CM3050 S (Leica, Wetzlar, Germany) cryostat. Sections were fixed with acetone for 10 min at RT and blocked with 5% BSA with 3% goat serum for 30 min at RT. Primary antibodies were incubated overnight at 4°C as indicated in Table S8. For detection, Alexa Flour 488- or 568-conjugated secondary antibodies (Invitrogen, Waltham, MT) diluted 1:500 were added for 1 h at RT followed by nuclei counterstain with Hoechst 33342 dye (Invitrogen). Images were acquired with the LSM700 (Carl Zeiss, Oberkochen, Germany) laser scanning confocal microscope and a DMI6000 fluorescent microscope (Leica). Images were processed with Fiji.

### Statistical analysis

All experiments were performed in at least three biological repeats if not stated differently in the figure legend. Statistical calculation was done using unpaired Student’s t test and one-way or two-way analysis of variance. All statistical analyses and corresponding graphs were generated using GraphPad Prism Software (version 8). Graphs show mean ± SD or SEM.

## Data Availability

All available data have been included in the manuscript.

## Supplemental information

Document S1. Figures S1–S14 and Tables S1, S4, S5, S7, and S8

Table S2. Off-targets predicted by CRISPOR (see excel table)

Table S3. Off-targets predicted by CrispRGold (see excel table)

Table S6. Primer used in the study (see excel table)

Document S2. Article plus supplemental information
