# Supplementary material for: Cas9-induced single cut enables highly efficient and template-free repair of a muscular dystrophy causing founder mutation
Source: Mol Ther Nucleic Acids. 2023 Feb 5;31:494–511. doi: 10.1016/j.omtn.2023.02.005 (PMC9972404; doi:10.1016/j.omtn.2023.02.005)
Supplement: Document S1. Figures S1–S14 and Tables S1, S4, S5, S7, and S8 [file mmc1.pdf]

OMTN, Volume *31*

Supplemental information

Cas9-induced single cut enables highly efficient and template-free repair of a muscular dystrophy causing founder mutation

Stefanie Müthel, Andreas Marg, Busem Ignak, Janine Kieshauer, Helena Escobar, Christian Stadelmann, and Simone Spuler

Supplemental Figures

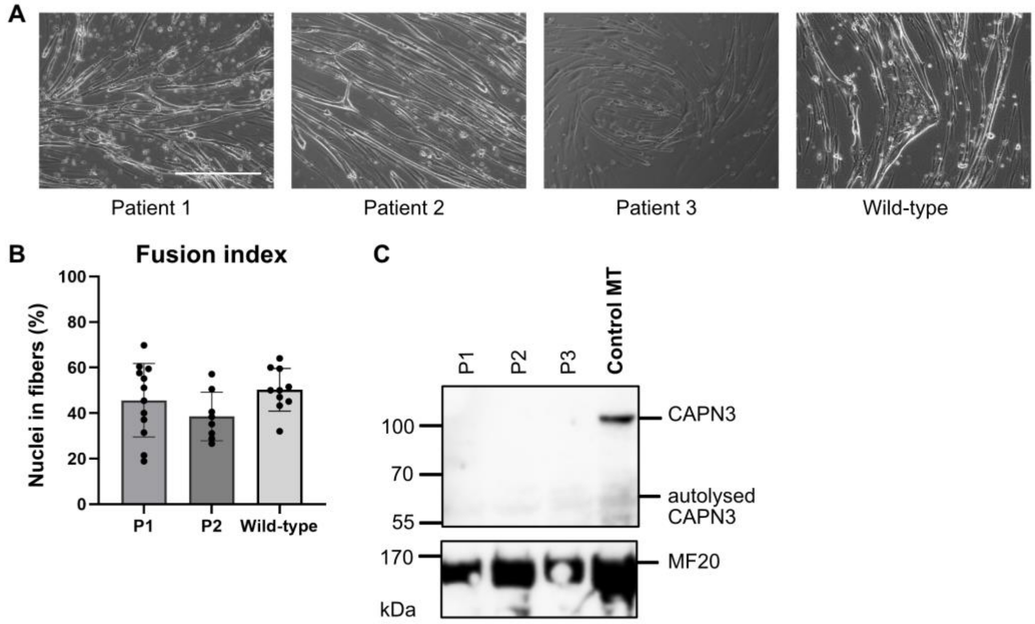

Figure S1. Differentiation into myotubes. (A) Differentiation potential of control and patient cell populations. All cell lines can differentiate into myotubes. Scale bar = 400 µm. (**B)** Fusion index of PHSats from patients 1 and 2 with *CAPN3* c.550delA$^{+/+}$. (**C)** Western blot to detect CAPN3 protein in differentiated myotubes. After full differentiation, no Calpain 3 is detected in myotubes from patients 1, 2 and 3. MF20 was used as loading and differentiation control. Control MT are derived from healthy controls.

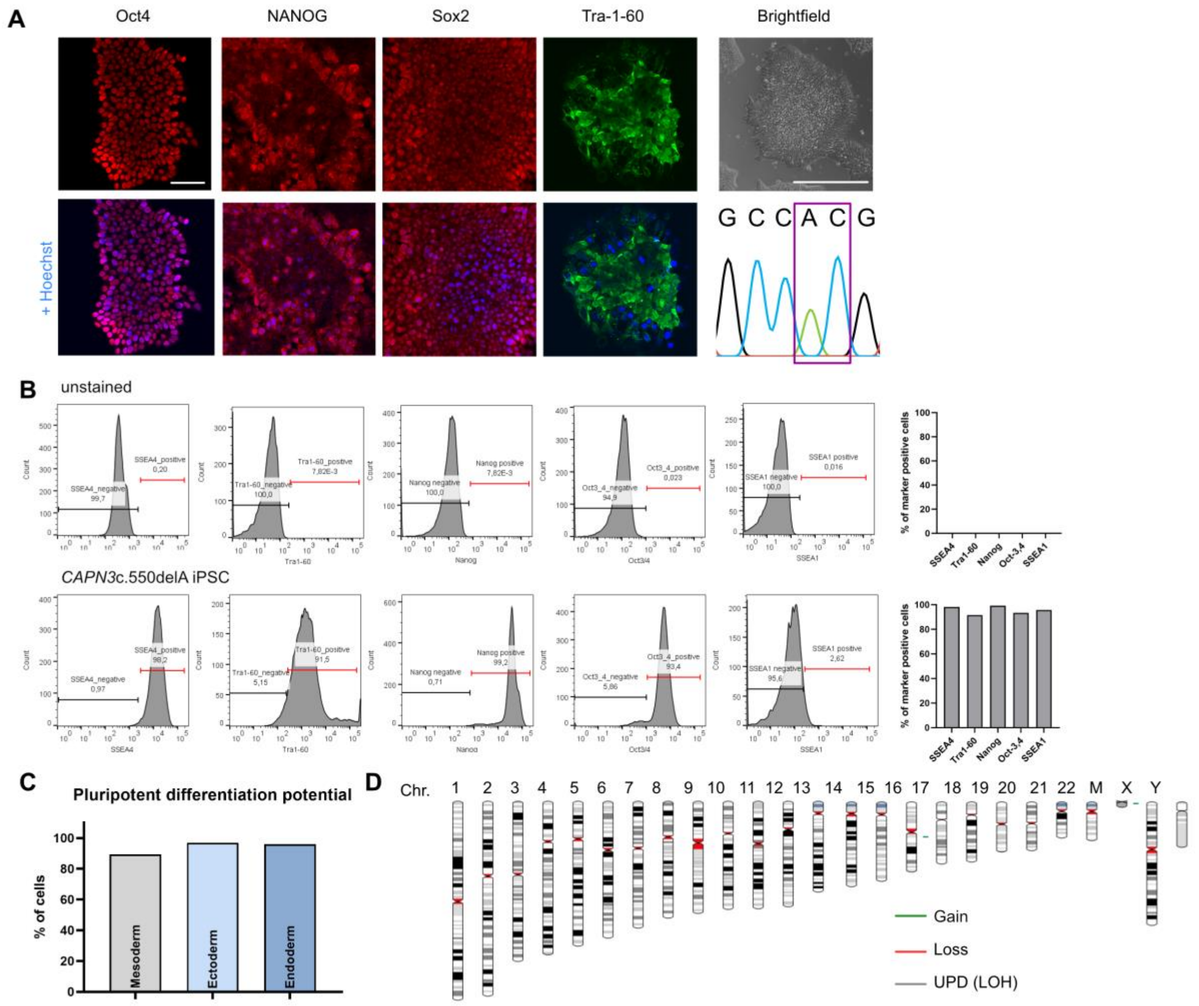

Figure S2. Generation and characterization of hiPSCs from patient PHSats. (A) Immunostaining of hiPSCs for pluripotency markers. Scale bar = 50 µm. A brightfield picture shows the morphology of hiPSCs (scale bar = 400 µm) and sequencing chromatogram shows *CAPN3* c.550delA mutation (purple square). **(B)** Histogram plot of flow cytometric analysis of the pluripotency markers SSEA4, Tra-160, NANOG, Oct4 and SSEA1. Top: Unstained cells as negative control. Bottom: Stained hiPSCs. Cells are positive for pluripotency markers. **(C)** hiPSCs can differentiate into mesoderm (CD140b, CD144), endoderm (Sox17, CD184), and ectoderm (Sox2, Pax6) lineage as analyzed by FACS. (**D)** Virtual karyotype analysis in patient hiPSCs. Green, gain (duplications); red, loss (deletions); grey, regions of uniparental disomy (loss of

heterozygosity). Reportable are copy number changes (gains and losses) greater than 0,4 Mb and regions of loss of heterozygosity above 3 Mb.

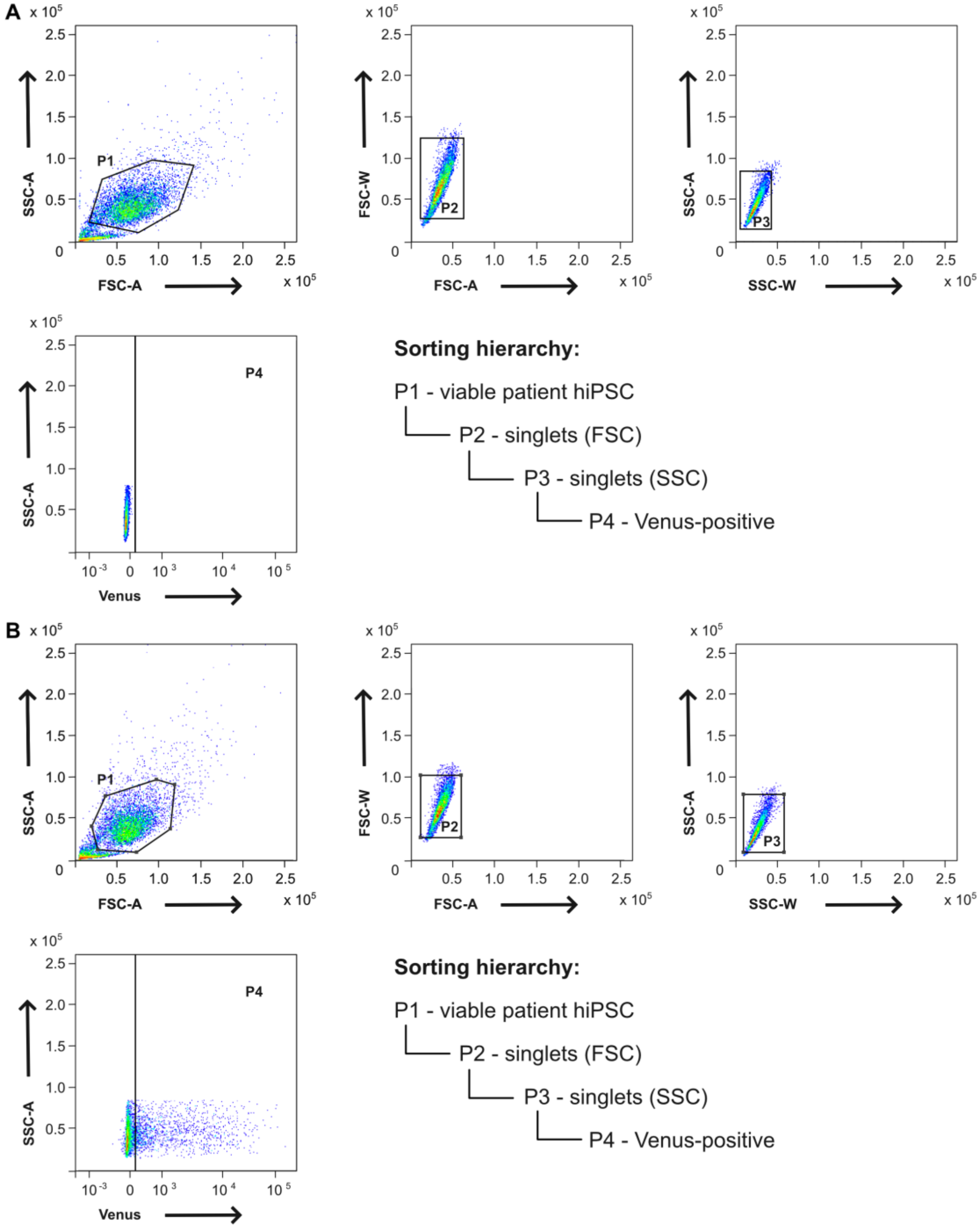

Figure S3. Gating strategy for FACS-sorting of Venus-positive hiPSCs. Representative FACS plots of hiPSCs transfected with either mock **(A)** or SpCas9::Venus and sgRNA **(B)** Gates were

defined to select viable hiPSCs (P1) and for doublet exclusion (P2, P3). Venus-positive events (P4) were separated by green fluorescence intensity.

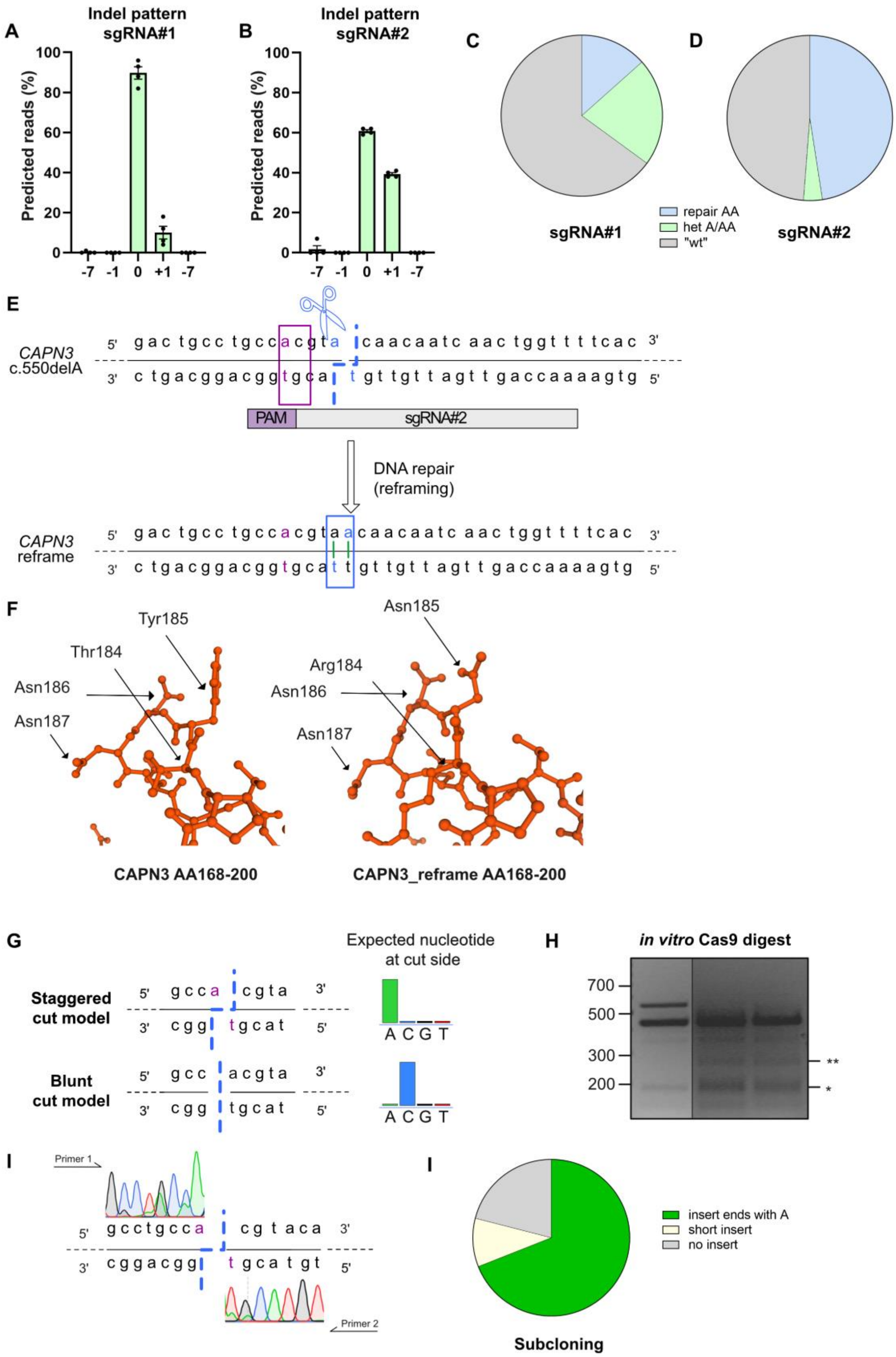
A
Indel pattern sgRNA#1
Predicted reads (%)
-7 -1 0 +1 -7
B
Indel pattern sgRNA#2
Predicted reads (%)
-7 -1 0 +1 -7
C
sgRNA#1
D
sgRNA#2
repair AA
het A/AA
"wt"
E
CAPN3 c.550delA
5' g a c t g c c t g c c a c g t a c a a c a a t c a a c t g g t t t t c a c 3'
3' c t g a c g g a c g g t g c a t g t t g t t a g t t g a c c a a a a g t g 5'
PAM
sgRNA#2
DNA repair (reframing)
CAPN3 reframe
5' g a c t g c c t g c c a c g t a a c a a c a a t c a a c t g g t t t t c a c 3'
3' c t g a c g g a c g g t g c a t t g t t g t t a g t t g a c c a a a a g t g 5'
F
Tyr185
Thr184
Asn186
Asn187
CAPN3 AA168-200
Asn185
Arg184
Asn186
Asn187
CAPN3_reframe AA168-200
G
Staggered cut model
5' g c c a c g t a 3'
3' c g g t g c a t 5'
Blunt cut model
5' g c c a c g t a 3'
3' c g g t g c a t 5'
Expected nucleotide at cut side
A C G T
A C G T
H
in vitro Cas9 digest
700
500
300
200
**
*
I
Primer 1
5' g c c t g c c a c g t a c a 3'
3' c g g a c g g t g c a t g t 5'
Primer 2
I
Subcloning
insert ends with A
short insert
no insert

Figure S4: ***Characterization of reframing with NHEJ.*** **(A)/(B)** Indel pattern of edited alleles for sgRNA#1 (A) and #2 (B). + 1 is the favored indel, n = 3. **(C)/(D)** Verification of predicted editing efficiency by analysis of single hiPSC colonies for sgRNA#1 (C) and #2 (D). Single colonies were picked and the DNA sequence of *CAPN3* was analyzed with Sanger sequencing. 48 colonies from three biological repeats were analyzed. **(E)** Potential mechanism of reframing with sgRNA#2. Top: DNA sequence of *CAPN3* c.550delA; purple square marks the position of the mutation, dotted blue line indicates the SpCas9 staggered cutting model. Bottom: Reframed *CAPN3* wild-type DNA sequence after repair of the DSB. **(F)** Prediction of the protein 3D structure after reframing with sgRNA#2 for AA168 - 200. The 3D structure of the protein is not severely changed. **(G)** Staggered cut and blunt end cut model for Cas9. In the staggered cut model, the expected nucleotide at the cut side is A, whereas it is C in the blunt end cut model. **(H)** Agarose gel of *in vitro* Cas9 digest. *, ** = bands of interest. **(I)** Sanger sequencing reads to investigate the Cas9 cut model after subjecting a PCR product from the *CAPN3* c.550delA locus with Cas9 and sgRNA as RNP. The nucleotide at the cut side is A. **(J)** Verification of the cutting pattern via subcloning. 54 bacterial colonies were analyzed.

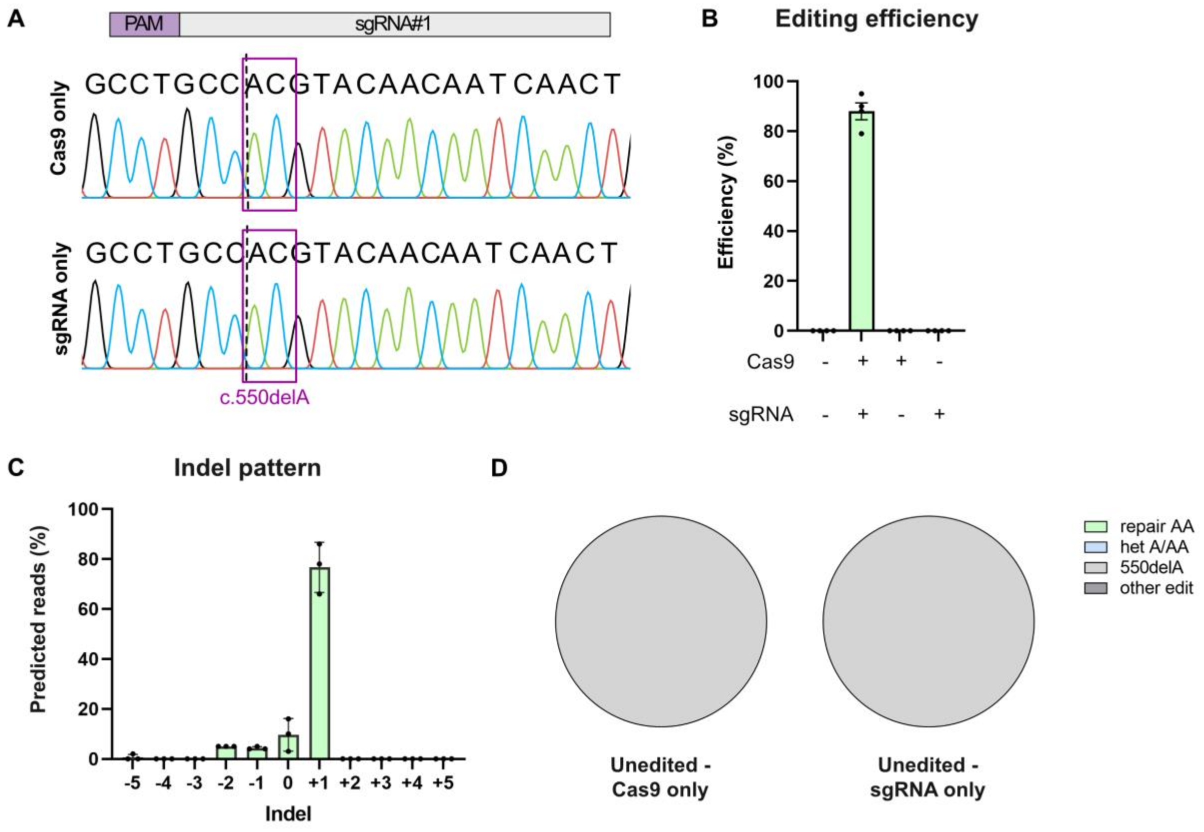

Figure S5. Efficient and precise reframing with mRNA in hiPSCs. (A) Representative Sanger sequencing result for SpCas9 only (top) and sgRNA only (bottom) controls. No indels were detected. **(B)** Quantification of editing efficiency. Only if the cells are transfected with SpCas9 and sgRNA, editing can be detected with an efficiency of > 80%, n = 3. **(C)** Indel pattern of edited alleles. +1 is the favored indel, n = 3. **(D)** Verification of the editing efficiency by analysis of single colonies for SpCas9 and sgRNA only controls. Single colonies of hiPSC were picked and the DNA sequence of *CAPN3* was analyzed with Sanger sequencing. 144 colonies in 3 biological repeats were analyzed.

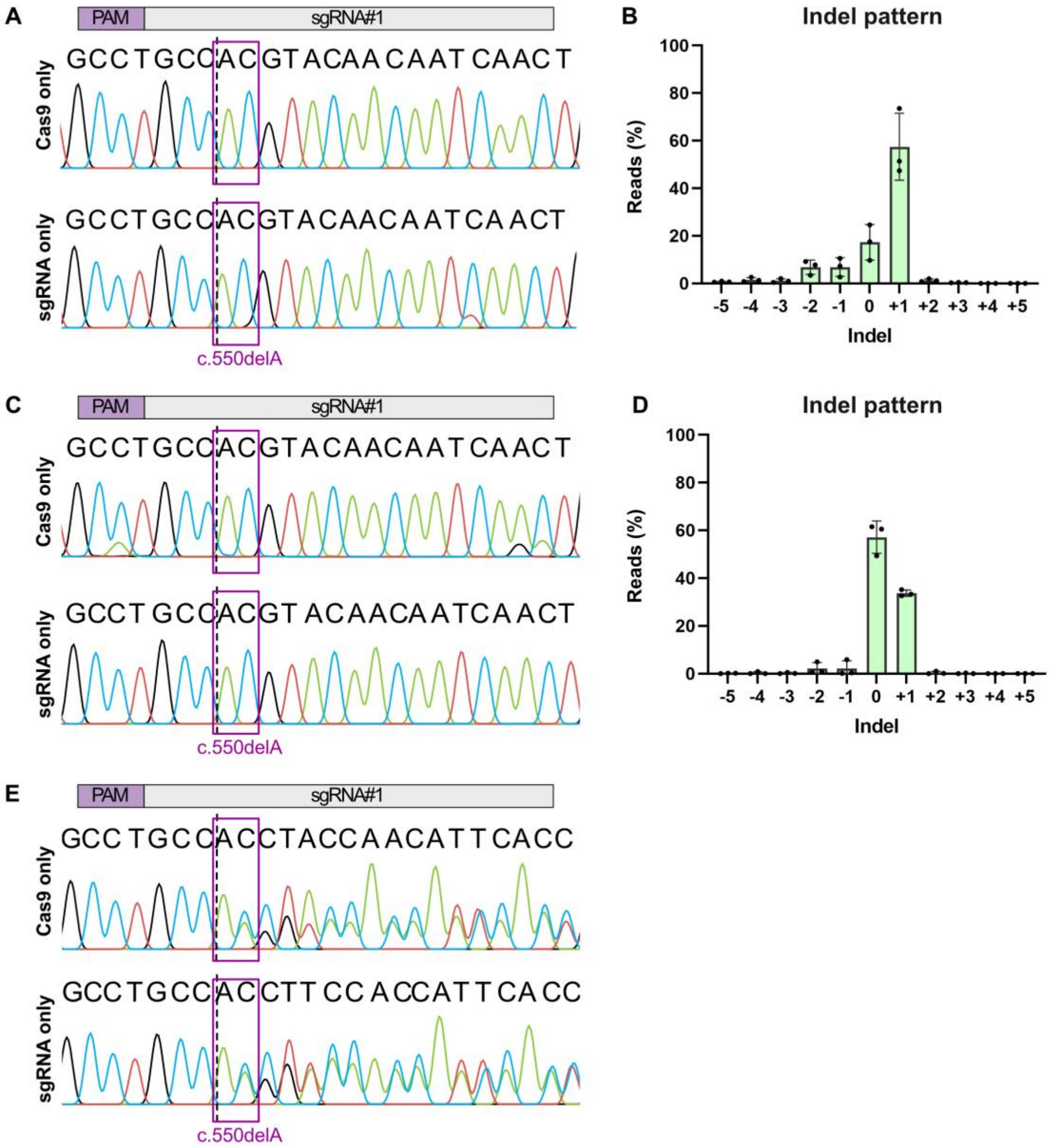

Figure S6. Efficient and precise reframing with mRNA including controls. (A) Sanger sequencing for SpCas9 only (top) and sgRNA (bottom) controls in PHSats from patient 1. No indels were detected. **(B)** Indel pattern of edited cells from patient 1. The indel pattern was calculated with CRISPResso2 after amplicon sequencing of 3 biological repeats. +1 is the favored indel. **(C)** Sanger sequencing for SpCas9 only and sgRNA controls in PHSats from patient 2. No indels were detected. **(D)** Indel pattern of edited cells from patient 2. The indel pattern was

calculated with CRISPResso2 after amplicon sequencing of 3 biological repeats. +1 is the favored indel. **(E)** Sanger sequencing for SpCas9 and sgRNA only controls in PHSats from compound heterozygous patient 3.

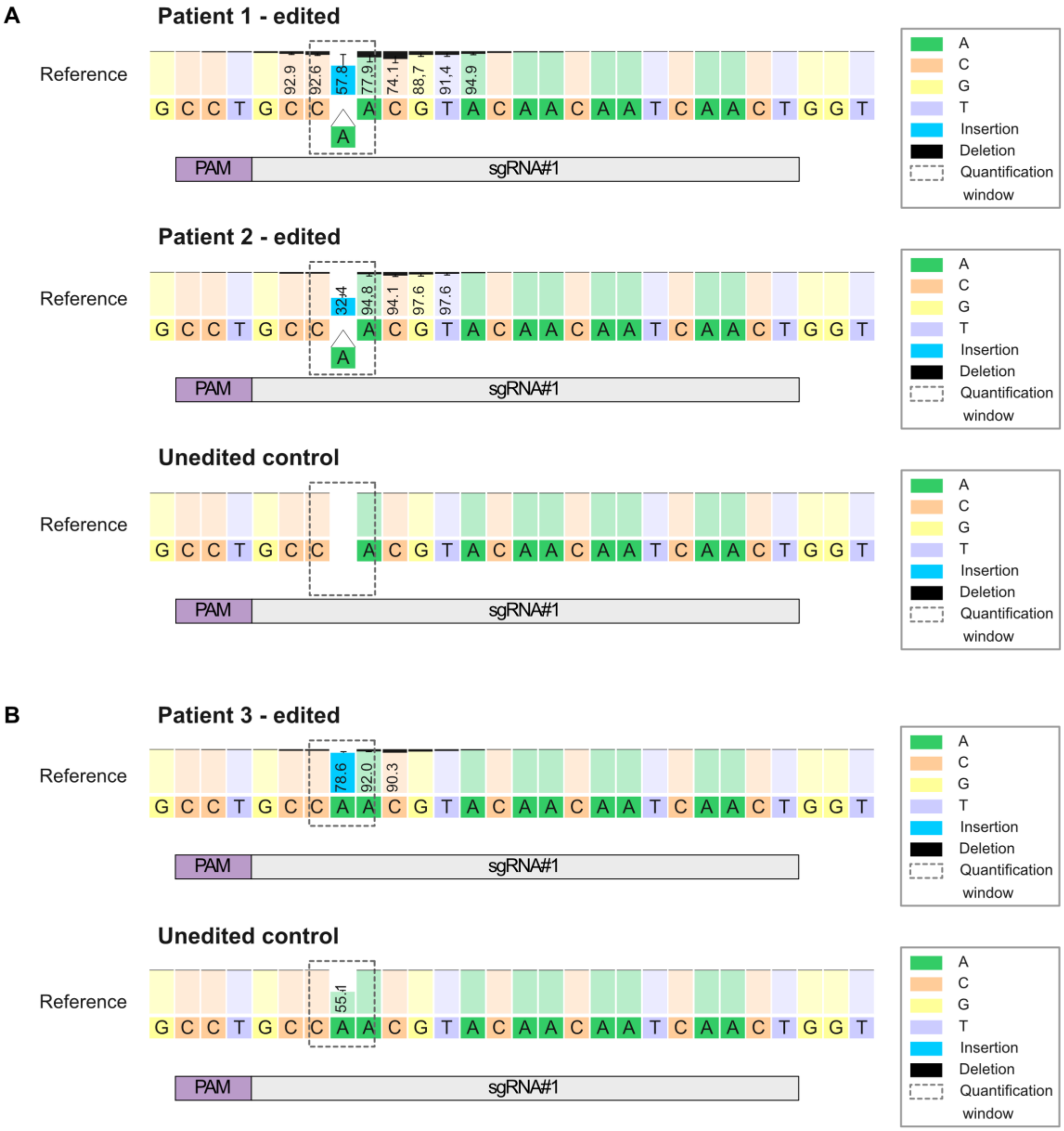

Figure S7. Nucleotide frequency across the amplicon. (A) Representative Crispresso2 analysis of the nucleotide frequency. Upon editing for both, patients 1 and 2, the nucleotide frequency changes with an insertion of one nucleotide in the quantification window. **(B)** Representative Crispresso2 analysis of the nucleotide frequency. Upon editing in patient 3 the nucleotide frequency of A at position of the mutation increases to 78,6 %.

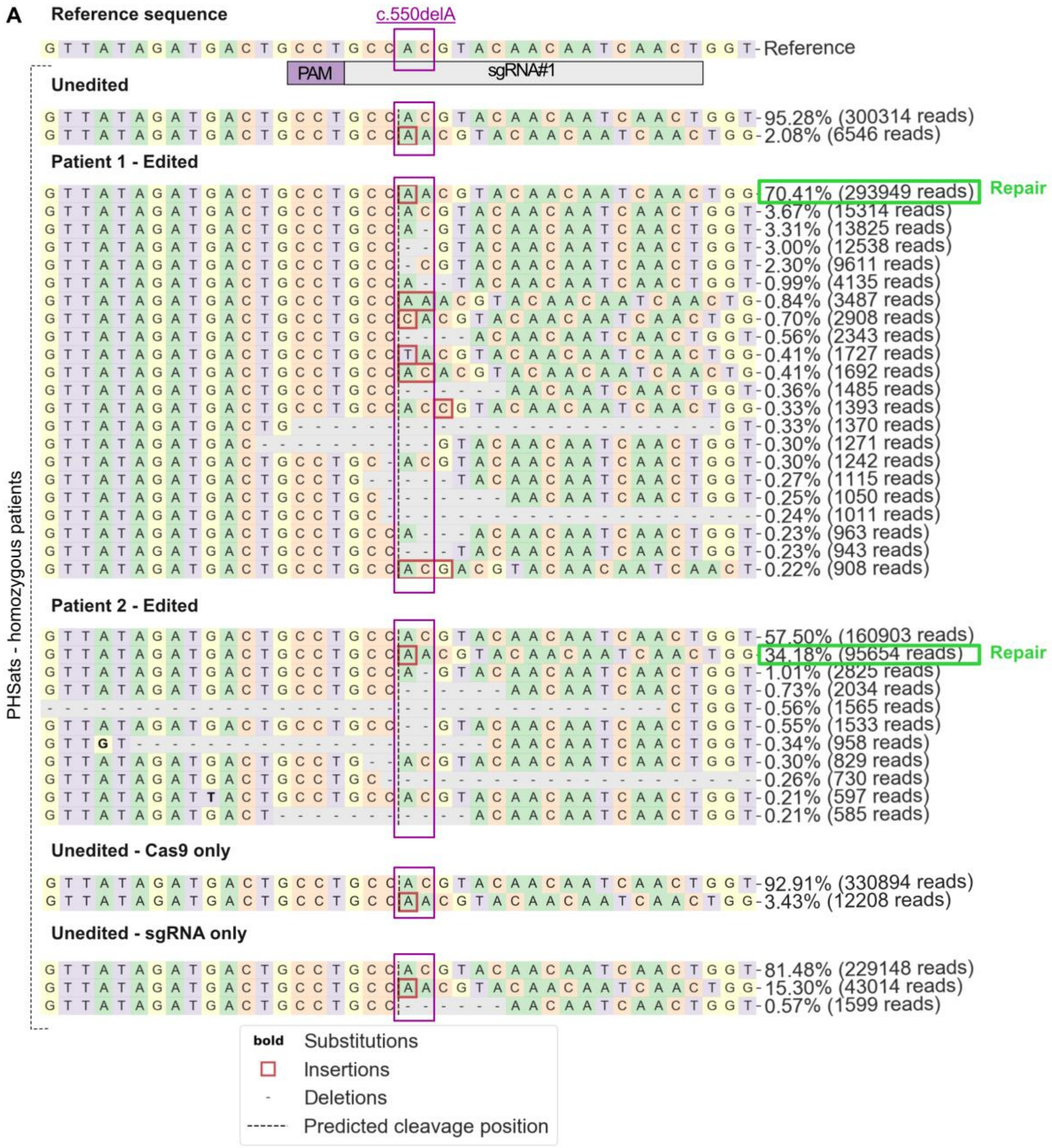

Figure S8. Allele frequencies after transfection of patients 1 and 2. (A) Representative Crispresso2 visualization of the distribution of identified alleles around the cleavage side. Purple box indicates the position of c.550delA. Substitutions are shown in bold, red bold rectangles highlight inserted sequences. Green box marks repair with + 1 insertion. Unedited controls are representatively shown from patient 1.

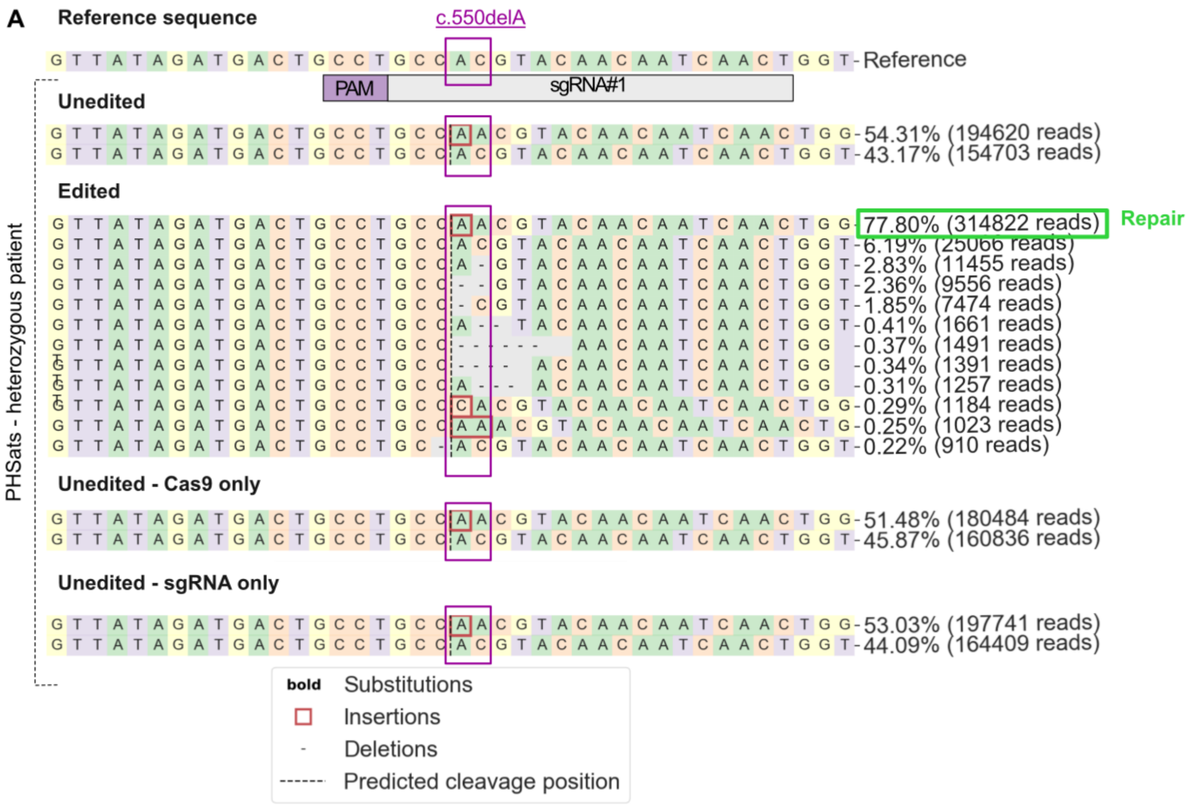

Figure S9. Allele frequencies after transfection of patient 3. (A) Crispresso2 visualization of the distribution of identified alleles around the cleavage side. Purple box indicates the position of c.550delA. Substitutions are shown in bold, red bold rectangles highlight inserted sequences. Green box marks repair with + 1 insertion.

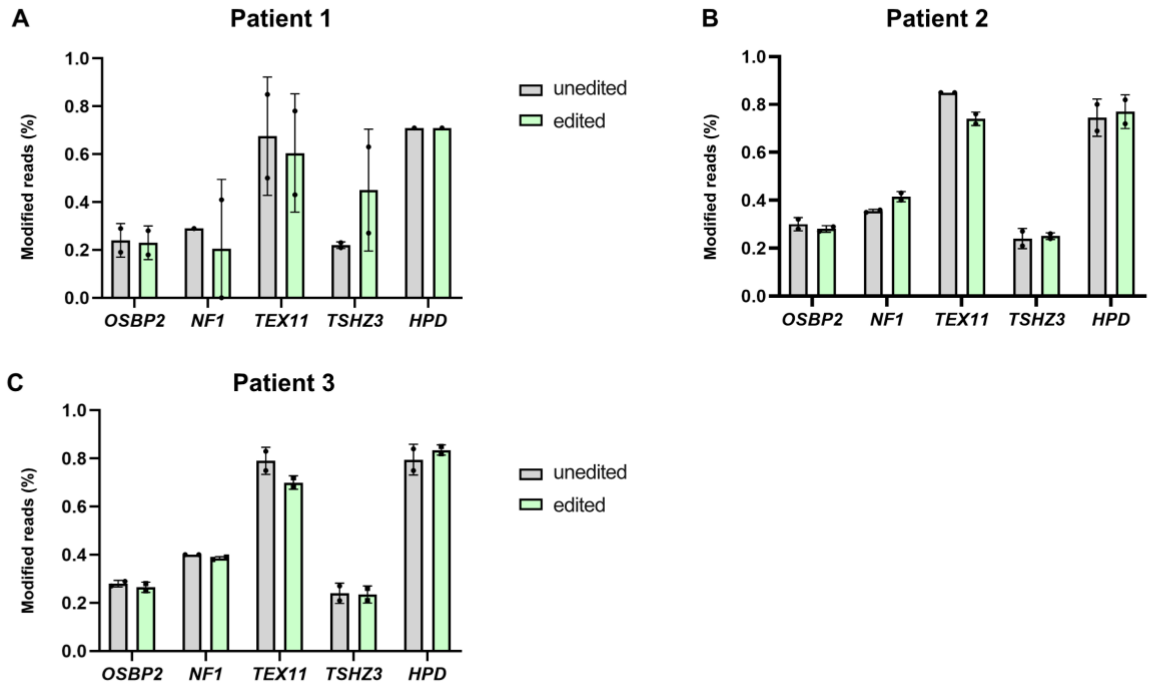

Figure S10. Off-target analysis. (A)/(B)/(C) Modified reads of the high potential and exonic off-targets for patients 1 (A), 2 (B) and 3 (C) that were edited or unedited. None of the off-targets shows a higher amount of modified reads in the edited vs. the unedited control for any of the genes. 2 biological repeats were analyzed.

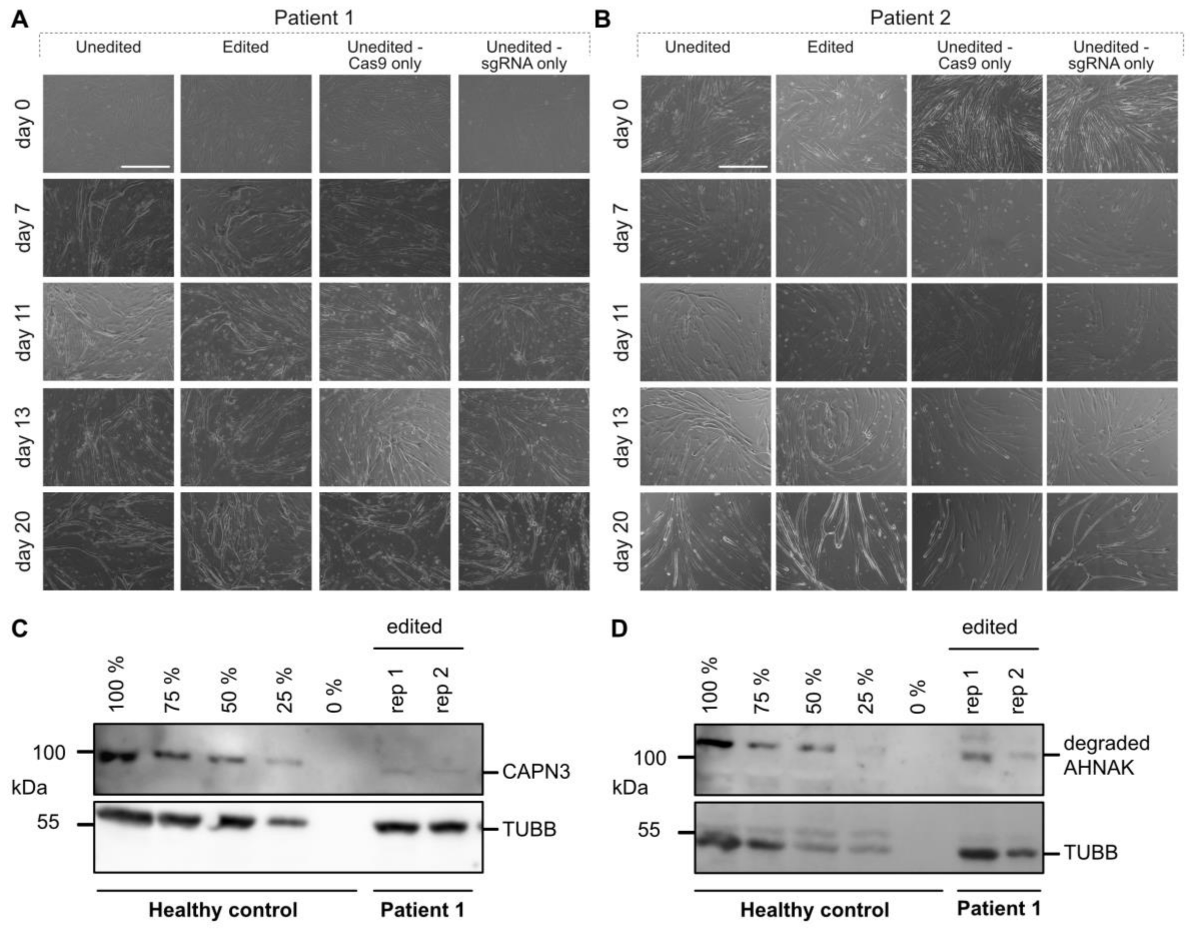

Figure S11. Myogenic properties of PHSats after gene editing. **(A)/(B)** Morphology of PHSats after gene editing compared in a 20-week time course experiment with subsequent differentiation into terminal myotubes. (A) patient 1, (B) patient 2. Scale bar = 400 µm. **(C)** Western Blot analysis of CAPN3 levels in myotubes from patient 1 compared to healthy control myotubes at different percentages of protein. **(D)** Western Blot analysis of degraded AHNAK in myotubes from patient 1 compared to healthy control myotubes at different percentages of protein.

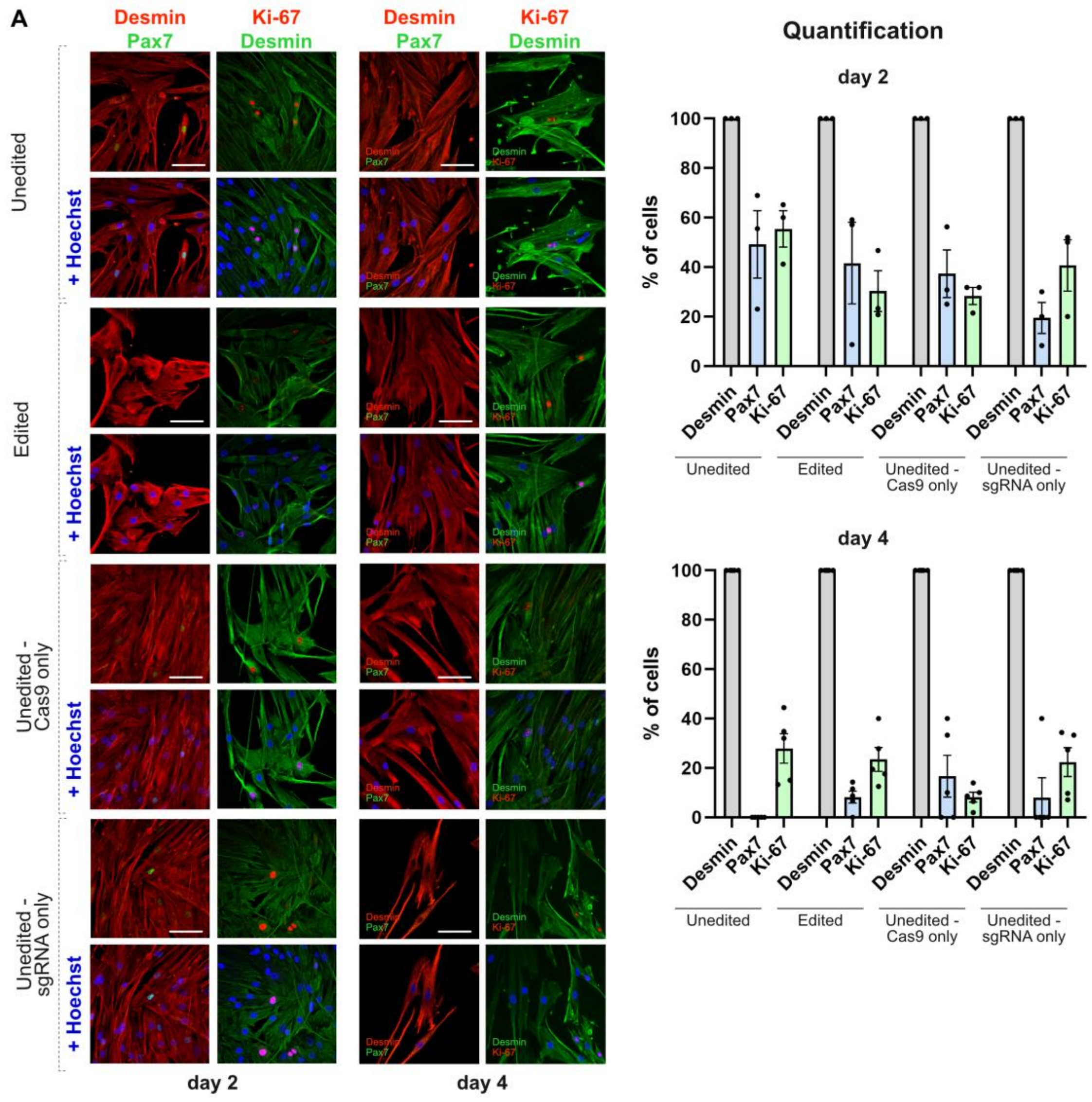

Figure S12. Myogenic properties of PHSats after gene editing (A) Left: Immunofluorescence staining for the myogenic marker Desmin, Pax7 and the proliferation marker Ki-67 at day 2 and 4 after transfection. Nuclei were counterstained with Hoechst. Right: Quantification of the immunofluorescence. At least 50 nuclei were counted for each condition: mean ± SEM of ≥ 3 images.

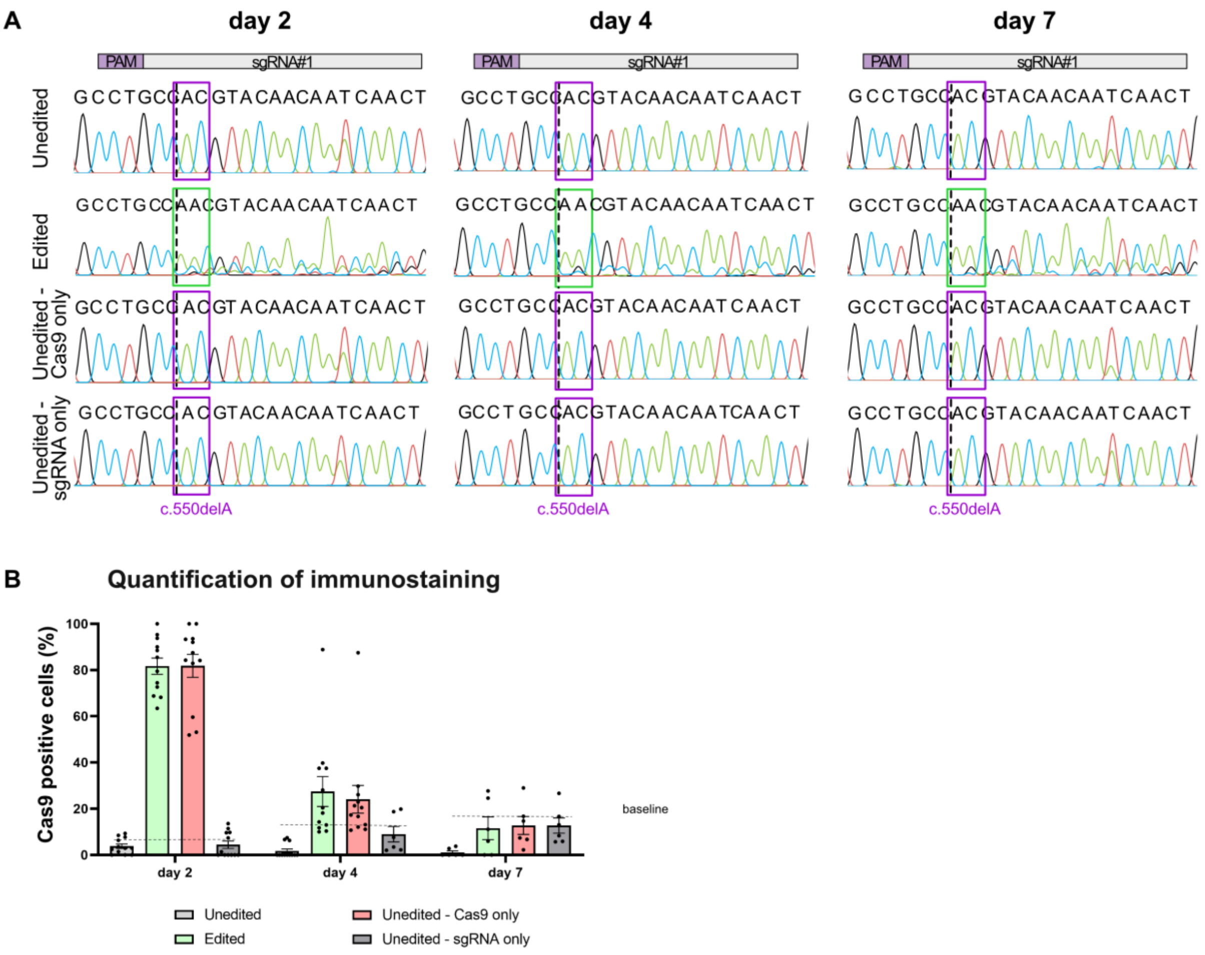

Figure S13. Rapid decrease in SpCas9 expression after nucleofection. (A) Sanger sequencing results at day 2, 4 and 7 after transfection of PHSats from patient 1. Binding site of the sgRNA is indicated on top, the purple square marks the position of the mutation, and the dotted line represents the cutting site of SpCas9. **(B)** Quantification of the immunofluorescence for SpCas9. At least 100 nuclei were counted for each condition: mean ± SEM of ≥ 5 images.

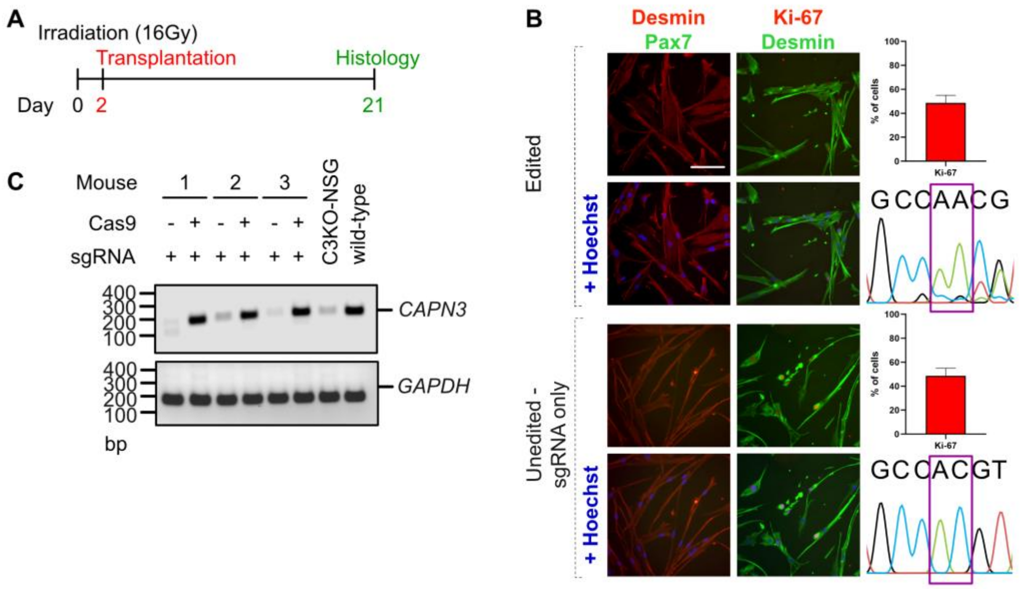

Figure S14. Transplantation of repaired PHSats into immune- and Calpain 3-deficient mice. **(A)** Experimental outline of transplantation experiments. Mice are irradiated and edited as well as unedited cells are injected into the TA muscle 2 days after. 21 days after transplantation, animals were sacrificed and according muscles analyzed. **(B)** Left: Immunostaining of PHSats for the myogenic marker Desmin and the proliferation marker Ki-67 from edited and unedited control cells from patient 1 that were used for transplantation. Nuclei were counterstained with Hoechst, scale bar = 130 µm. At least 150 cells were counted. Right: Quantification of Ki-67 and DNA-sequence at the position of the mutation c.550delA. Edited cells show wild-type DNA sequence. **(C)** RT-PCR analysis of CAPN3 expression after transplantation. Only muscles transplanted with edited cells show CAPN3 expression.

Table S1. Clinical information on PHSats donors

Donor ID	Age at biopsie	Gender	Muscle Histology	CAPN3 mutation
1	43	m	dystrophic	c.550delA; c.550delA
2	18	m	mildly dystrophic	c.550delA; c.550delA
3	15	m	mildly dystrophic	c.550delA; c.598_612del
Control	13	m	normal	no

Table S2. Off-targets predicted by CRISPOR (see excel table)

Table S3. Off-targets predicted by CrispRGold (see excel table)

Table S4. Overview of engraftment after transplantation

		Max. amount of human muscle fibers after treatment with	
**Mouse**	**Engraftment**	**sgRNA only (left leg)**	**Cas9+sgRNA (right leg)**
1	yes	0	35
2	yes	8	6
3	yes	0	8
4	yes	0	15
5	yes	29	7
6	yes	8	29
7	yes	4	34

Table S5. sgRNA used in this study

**Name**	**Sequence 5‘ > 3‘** (crRNA + tracrRNA)	**Target**
sgRNA#1	AGTTGATTGTTGTACGTGGCGUUUUAGAGCUAGAAAUAGCAAGUUAAAAUAAGGCUAGUCCGUUAUCAACUUGAAAAAGUGGCACCGAGUCGGUGCUUUU	*CAPN3* c.550delA

Table S6. Primer used in the study (see excel table)

Table S7. Components of Differentiation medium 2

**Component**	**Concentration**	**Manufacturer**
Neurobasal medium		Thermo Fisher Scientific, Waltham, MT, USA
B27 (50x)	1x	Thermo Fisher Scientific, Waltham, MT, USA
Glutamax (100x)	1x	Thermo Fisher Scientific, Waltham, MT, USA
Glial cell derived neutrophic factor (GDNF)	10 ng/ml	Peprotech, London, UK

**Component**	**Concentration**	**Manufacturer**
Brain-derived neutrophic factor (BDNF)	10 µg/ml	Peprotech, London, UK
Sonic Hedgehog (Shh)	50 ng/ml	R&D Systems, Minneapolis, MN, USA
Retinoic Acid	0,1 µM	Merck, Darmstadt, Germany
Insulin-like growth factor 1 (IGF-1)	10 ng/ml	Peprotech, London, UK
Ciliary neurotrophic factor (CNTF)	5 ng/ml	Peprotech, London, UK
Neurotrophin-3 (NT3)	20 ng/ml	Peprotech, London, UK
Neurotrophin-4 (NT4)	20 ng/ml	Peprotech, London, UK
Vitronection	100 ng/ml	Merck, Darmstadt, Germany
Mouse laminin	4 µg/ml	Thermo Fisher Scientific, Waltham, MT, USA
Agrin (100x)	100 ng/ml	R&D Systems, Minneapolis, MN, USA

Table S8. Antibodies used in this study

Antibody	Manufacturer (catalogue number)	Purpose	Working concentration
AHNAK	Abnove (Clone 3G7)	Western Blot	1:500
CAPN3 12A2	Novocastra (NCL-CALP-12A2)	Western Blot	1:50
Cas9	Novus Biologicals (NBP2-36440)	Western Blot	1:500
		Staining	1:500
Desmin	DAKO (M0760)	Staining	1:50
Desmin	Abcam (ab15200)	Staining	1:2,000
Ki-67	Thermo Fisher Scientific (MA5-14520)	Staining	1:200
Lamin A/C	Abcam (ab108595)	Staining	1:4,000
Nanog	Abcam (ab21624)	Staining	1:100
MF20	DSHB	Western Blot	1:2,000
Oct4	Abcam (ab19857)	Staining	1:1,000
Sox2	Abcam (ab97959)	Staining	1:300
Pax7	Santa Cruz (sc-81648)	Staining	1:200
Tra-1-60	Abcam (ab16288)	Staining	1:500
TUBB	Abcam (ab6046)	Western Blot	1:2,000
Spectrin	Novocastra (NCL-SPEC1)	Staining	1:100

Antibody	Manufacturer (catalogue number)	Purpose	Working concentration
Skeletal Myosin (Fast)	Sigma (M4276)	Staining (Fusion index)	1:100
Phalloidin-FITC	Sigma (49409)	Staining (Fusion index)	1:2,000
